# Characterisation of cytotoxicity and DNA damage induced by the topoisomerase II-directed bisdioxopiperazine anti-cancer agent ICRF-187 (dexrazoxane) in yeast and mammalian cells

**DOI:** 10.1186/1471-2210-4-31

**Published:** 2004-12-02

**Authors:** Lars H Jensen, Marielle Dejligbjerg, Lasse T Hansen, Morten Grauslund, Peter B Jensen, Maxwell Sehested

**Affiliations:** 1 Department of Pathology, Diagnostic Centre, Rigshospitalet 5444, Frederik V's Vej 11, DK-2100 Copenhagen, Denmark; 2 Institute of Molecular Pathology, University of Copenhagen, Frederik V's Vej 11, DK-2100, Copenhagen, Denmark; 3 Laboratory of Experimental Medical Oncology, Finsen Centre, Rigshospitalet 5074, Blegdamsvej 9, DK-2100 Copenhagen, Denmark

## Abstract

**Background:**

Bisdioxopiperazine anti-cancer agents are inhibitors of eukaryotic DNA topoisomerase II, sequestering this protein as a non-covalent protein clamp on DNA. It has been suggested that such complexes on DNA represents a novel form of DNA damage to cells. In this report, we characterise the cytotoxicity and DNA damage induced by the bisdioxopiperazine ICRF-187 by a combination of genetic and molecular approaches. In addition, the well-established topoisomerase II poison m-AMSA is used for comparison.

**Results:**

By utilizing a panel of *Saccharomyces cerevisiae *single-gene deletion strains, homologous recombination was identified as the most important DNA repair pathway determining the sensitivity towards ICRF-187. However, sensitivity towards m-AMSA depended much more on this pathway. In contrast, disrupting the post replication repair pathway only affected sensitivity towards m-AMSA. Homologous recombination (HR) defective irs1SF chinese hamster ovary (CHO) cells showed increased sensitivity towards ICRF-187, while their sensitivity towards m-AMSA was increased even more. Furthermore, complementation of the XRCC3 deficiency in irs1SF cells fully abrogated hypersensitivity towards both drugs. DNA-PK_cs _deficient V3-3 CHO cells having reduced levels of non-homologous end joining (NHEJ) showed slightly increased sensitivity to both drugs. While exposure of human small cell lung cancer (SCLC) OC-NYH cells to m-AMSA strongly induced γH2AX, exposure to ICRF-187 resulted in much less induction, showing that ICRF-187 generates fewer DNA double strand breaks than m-AMSA. Accordingly, when yeast cells were exposed to equitoxic concentrations of ICRF-187 and m-AMSA, the expression of DNA damage-inducible genes showed higher levels of induction after exposure to m-AMSA as compared to ICRF-187. Most importantly, ICRF-187 stimulated homologous recombination in SPD8 hamster lung fibroblast cells to lower levels than m-AMSA at all cytotoxicity levels tested, showing that the mechanism of action of bisdioxopiperazines differs from that of classical topoisomerase II poisons in mammalian cells.

**Conclusion:**

Our results point to important differences in the mechanism of cytotoxicity induced by bisdioxopiperazines and topoisomerase II poisons, and suggest that bisdioxopiperazines kill cells by a combination of DNA break-related and DNA break-unrelated mechanisms.

## Background

Type II topoisomerases are essential nuclear enzymes found in all living organisms [1]. Their basic role in cells is to catalyse the transport of one DNA double helix through a transient double strand break in another DNA molecule [2]. This activity helps relieve tensions built up in DNA during various DNA metabolic processes such as DNA replication, chromosome condensation and de-condensation, chromosome segregation and transcription [3].

Topoisomerase II is also a major drug target in human cancer therapy, where a number of clinically active drugs such as the epipodophyllotoxins VP-16 and VM-26, the aminoacridine m-AMSA, and antracyclines such as doxorubicin, daunorubicin and epirubicin are widely used. These drugs have collectively been called topoisomerase II poisons due to their mechanism of action on topoisomerase II. Rather than inhibiting the basic catalytic activity of the enzyme, these drugs perturb the topoisomerase II catalytic cycle resulting in an increase in the level of a transient reaction intermediate, where DNA is cleaved and covalently attached to DNA [4].

Catalytic inhibitors of topoisomerase II have a different mode of action. These drugs exemplified by merbarone, aclarubicin, F11782 and the bisdioxopiperazines work by inhibiting topoisomerase II at other stages in the reaction cycle where DNA is not cleaved as reviewed in [5,6]. Amongst these, the bisdioxopiperazines have gained much attention due to their distinct and well-characterised mode of action. These compounds exemplified by ICRF-187, ICRF-159 and ICRF-154 inhibit the DNA strand passage reaction of topoisomerase II by sequestering this protein as a salt-stable closed clamp on DNA whose formation depends on the presence of ATP [7-9]. This closed clamp complex has retained the capability to hydrolyse ATP, although at a reduced level [10].

Several studies indicate that the closed clamp complex on DNA represents a novel form of DNA lesion to cells, – and that inhibition of topoisomerase II catalytic activity (DNA strand passage activity) is not responsible for bisdioxopiperazine-induced cell kill: (*i*) Expression of bisdioxopiperazine-sensitive topoisomerase II in cells also expressing bisdioxopiperazine-resistant topoisomerase II confers dominant sensitivity to these drugs [7,11] – a modality reminiscent of that of topoisomerase II poisons. (*ii*) Mouse embryonic stem cells [12] and chicken lymphoma DT40 cells [13] having one topoisomerase II α allele knocked out with concomitant reduced levels of topoisomerase II, are resistant to both ICRF-193 and the topoisomerase II poison etoposide, – while the opposite result is to be expected if ICRF-193 kill cells by depriving them of essential topoisomerase II catalytic activity. (*iii*) Killing of yeast cells by exposure to ICRF-193 occurs more rapidly and to a higher level than killing of yeast cells induced by the depletion of endogenous topoisomerase II catalytic activity [7]. (*iv*) The ICRF-193-induced topoisomerase II closed clamp complexes on DNA work as a "road block" signalling selective degradation of topoisomerase II β as well as p53 activation in a transcription dependent fashion [14].

Some studies have recorded elevated levels of DNA breaks in cells after exposure to the bisdioxopiperazine analog ICRF-193. In one study, ICRF-193 was found to increase the level of topoisomerase II-DNA covalent complexes *in vitro *and *in vivo *[15]. However, in this study efficient trapping of this covalent intermediate was only evident when guanidine was used to denature topoisomerase II attracted to DNA, while the agent normally used to trap the topoisomerase II-DNA cleavage complex, SDS, was not effective. In another study, the comet assay and pulsed field gel electrophoresis were used to demonstrate elevated levels of DNA breaks in mammalian cells after exposure to ICRF-193 [16]. In this study, inhibiting DNA replication with aphidicolin reduced the level of DNA breaks induced by the topoisomerase II poison m-AMSA, but had no effect on DNA breaks induced by ICRF-193. These results point towards bisdioxopiperazines poisoning DNA topoisomerase II in cells by a mechanism different from that of the classical topoisomerase II poisons such as etoposide and m-AMSA. In a recent paper, it was directly demonstrated that m-AMSA-induced dominant cytotoxicity only required the DNA cleavage activity of topoisomerase II, while dominant cytotoxicity towards ICRF-193 depended strictly on the DNA strand passage reaction of the enzyme[17].

Based on these observations, the present study aims to further elucidate the mechanism of cytotoxicity induced by the bisdioxopiperazines. We here characterise the effect of the clinically approved analog ICRF-187 (dexrazoxane) by using a number of different cell-based pharmacological assays, taking advantage of genetically modified yeast and mammalian cells.

## Results

### ICRF-187 sensitivity of yeast cells depends on their homologous recombination status, albeit to a lesser extent than for m-AMSA sensitivity

To pinpoint the mechanism of cytotoxicity of ICRF-187 versus m-AMSA, we employed a panel of human topoisomerase II α-transformed haploid single-gene knockout yeast strains, defective in various aspects of DNA repair, checkpoint control, membrane transport and protein degradation. All yeast strains are depicted in table 1. We used doses of these two drugs equitoxic to wild-type cells having no mutations. Clonogenic survival of all yeast strains is depicted in additional file 1, and the degree of drug resistance / hypersensitivity is also listed in table 2.

**Table 1:** Yeast strains used in the study

BY4741 + pMJ1	BY4741Δ*rad9 *+ pMJ1
BY4741Δ*rad51 *+ pMJ1	BY4741Δ*tel1 *+ pMJ1
BY4741Δ*rad52 *+ pMJ1	BY4741Δ*chk1 *+ pMJ1
BY4741Δ*rad54 *+ pMJ1	BY4741Δ*mhl1 *+ pMJ1
BY4741Δ*rad55 *+ pMJ1	BY4741Δ*pms1 *+ pMJ1
BY4741Δ*rad57 *+ pMJ1	BY4741Δ*msh2 *+ pMJ1
BY4741Δ*rad59 *+ pMJ1	BY4741Δ*msh3 *+ pMJ1
BY4741Δ*dcm1 *+ pMJ1	BY4741Δ*atr1 *+ pMJ1
BY4741Δ*sae2 *+ pMJ1	BY4741Δ*pdr5 *+ pMJ1
BY4741Δ*rad50 *+ pMJ1	BY4741Δ*yor1 *+ pMJ1
BY4741Δ*mre11 *+ pMJ1	BY4741Δ*ubc4 *+ pMJ1
BY4741Δ*xrs2 *+ pMJ1	BY4741Δ*ubc13 *+ pMJ1
BY4741Δ*rad6 *+ pMJ1	BY4741Δ*doa4 *+ pMJ1
BY4741Δ*rad18 *+ pMJ1	BY4741Δ*qri8 *+ pMJ1
BY4741Δ*rev1 *+ pMJ1	BY4741Δ*rnr3 *+ pMJ1
BY4741Δ*rev3 *+ pMJ1	BY4741Δ*sml1 *+ pMJ1
BY4741Δ*rad1 *+ pMJ1	BY4741 + PYX112
BY4741Δ*rad14 *+ pMJ1	BY4741Δ*rad6 *+ pYX112
BY4741Δ*apn1 *+ pMJ1	BY4741Δ*rad50 *+ pYX112
BY4741Δ*yku70 *+ pMJ1	BY4741Δ*rad52 *+ pYX112
BY4741Δ*yku80 *+ pMJ1	BY4741Δ*sae2 *+ pYX112
BY4741Δ*mec3 *+ pMJ1	BY4741Δ*yku70 *+ pYX112
BY4741Δ*dcc1 *+ pMJ1	
BY4741Δ*rad17 *+ pMJ1	JN362A_t2-4_+ pMJ1

**Table 2:** Hypersensitivity (or resistance) scoring of pMJ1-transformed BY4741 deletion strains towards ICRF-187 and m-AMSA as determined in clonogenic assay using 22.5 hours drug exposure.

**Drug**	**ICRF-187**	**m-AMSA**
Gene deleted		
**WT**	0	0
**Nucleotide Excision Repair (NER)**		
**Single Strand Annealing (SSA) Recombination**		
**Anti-Recombination**		
Δ*rad1*	R	0
Δ*rad14*	0	0
**Mismatch Repair (MMR)**		
**Anti-Recombination**		
Δ*msh2*	R	0
Δ*msh3*	0	0
Δ*mhl1*	R	0
Δ*pms1*	R	0
**Base Excision Repair (BER)**		
Δ*apn1*	0	0
**Post Replication Repair (PRR)**		
Δ*rev1*	0	0
Δ*rev3*	0	0
Δ*rad18*	0	+
Δ*rad6*	0	++
**Homologous Recombination (HR)**		
**Non-Homologous End Joining (NHEJ)**		
Δ*rad50*	+	++
Δ*mre11*	+	++
Δ*xrs2*	+	++
**Homologous Recombination (HR)**		
Δ*rad52*	+	++
Δ*rad51*	+	+
Δ*rad54*	+	++
Δ*rad57*	+	++
Δ*rad55*	+	++
Δ*rad59*	0	0
Δ*dmc1*	0	0
Δ*sae2*	+	+
**Non-Homologous End Joining (NHEJ)**		
Δ*yku70*	+	0
Δ*yku80*	0	0
**DNA Damage Checkpoints**		
Δ*tel1*	0	0
Δ*rad9*	0	0
Δ*mec3*	0	0
Δ*ddc1*	0	0
Δ*rad17*	0	0
Δ*chk1*	0	0
**ABC Transporters (Yeast MDR1 homologous)**		
Δ*atr1*	0	0
Δ*pdr5*	+	0
Δ*yor1*	0	0
**Ubiquitin conjugation / hydrolysis**		
Δ*ubc4*	0	0
Δ*ubc13*	0	0
Δ*doa4*	0	R
Δ*qri8*	0	0
**Ribonucleotide-reductase regulation**		
Δ*rnr3*	0	0
Δ*sml1*	0	0

R : Cells are more than a 1/2 log resistant at any drug concentration.

0 : Cells are no more than a 1/2 log resistant and no more than a 1/2 log hypersensitive at any drug concentration.

+ : Cells are at least a 1/2 log but no more than 2 log hypersensitive at any concentration.

++ : Cells are more than 2 log hypersensitive at any concentration.

Hypersensitivity (resistance) was graduated as follows

The products of the three genes *RAD50*, *MRE11 *and *XRS2 *together form the Rad50/Mre11/Xrs2 hetero-trimer protein complex that has catalytic and structural functions in many kinds of DNA metabolic processes including HR as reviewed in [18]. We observed that Δ*rad50*, Δ*mre11*, and Δ*xrs2 *single knockout strains were extremely hypersensitive towards m-AMSA, while they displayed considerably less hypersensitivity towards ICRF-187 (additional file 1 and table 2).

We also tested the effect of deleting a number of genes exclusively involved in HR namely *RAD51*, *RAD52*, *RAD54*, *RAD55*, *RAD57*, *RAD59*, *DMC1 *and *SAE2 *[18]. Deleting *RAD51*, *RAD52*, *RAD54*, *RAD55*, *RAD57 *and *SAE2 *had a profound effect on the sensitivity of the yeast cells towards m-AMSA while having a smaller, but significant, effect on the sensitivity of these cells towards ICRF-187 (additional file 1 and table 2), again pointing to the HR pathway as being most important for the repair of DNA damage caused by cleavage complex stabilising drugs. We found that deleting *RAD59 *had no effect on drug sensitivity, confirming reported data that *RAD59 *only becomes functionally important in the absence of functional Rad51 protein [19]. We also observed no effect of deleting *DMC1 *(additional file 1 and table 2). This may be explained by the fact that Dmc1p is primarily involved in meiotic recombination [20].

NHEJ represents another DNA repair-pathway. In yeast, this repair pathway is generally less important than HR for the repair of DNA breaks [21]. In accordance with this we observed no effect of deleting the NHEJ genes *YKU70 *and *YKU80 *on the sensitivity towards m-AMSA. We did however, observe some hypersensitivity of Δ*yku70 *cells towards ICRF-187, while Δ*yku80 *cells were not hypersensitive (additional file 1 and table 2). This is a surprising result, because Yku70p and Yku80p have been demonstrated to play equally important roles for NHEJ activity in yeast [21]. These results suggest that the effect of deleting *YKU70 *is unrelated to its DNA repair functions.

**Figure 1:**
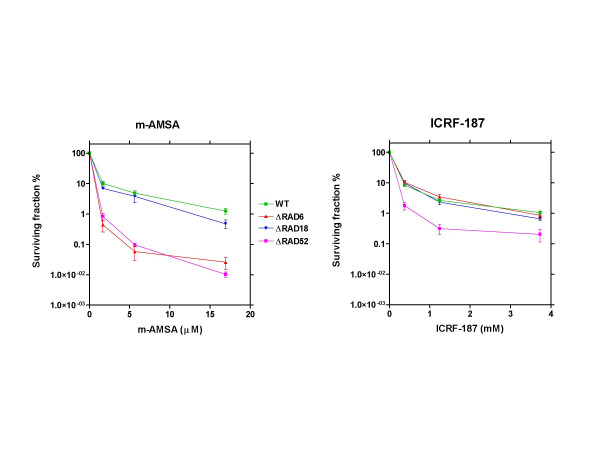
Clonogenic sensitivity of PRR defective Δ*rad6 *and Δ*rad18 *yeast cells towards equitoxic doses of ICRF-187 and m-AMSA. A Δ*rad52 *strain is included for comparison. Error-bars represent SEM of at least 3 experiments.

The DNA binding Rad18p forms a hetero-dimer with Rad6p that is involved in post replication repair (PRR) [22]. We found that although Δ*rad18 *cells were clearly hypersensitive towards m-AMSA, Δ*rad6 *cells were markedly more sensitive. Δ*rad6 *cells were actually among the most sensitive towards m-AMSA (figure 1, additional file 1 and table 2). Interestingly, the sensitivity of Δ*rad6 *and Δ*rad18 *cells towards ICRF-187 is indistinguishable from that of wild-type cells (figure 1). The vast difference in the sensitivity of Δ*rad*6 cells towards ICRF-187 and m-AMSA confirms the notion that the DNA lesions induced by these drugs are different in nature. The finding that Δ*rad6 *cells are much more sensitive towards m-AMSA than Δ*rad18 *cells is surprising, and may indicate that Rad6p functions unrelated to DNA repair affect cellular sensitivity towards m-AMSA. Rad6p has ubiquitin conjugating activity [22], and therefore such Rad18p-unrelated functions could involve protein degradation via the 26S proteasome pathway. To test this hypothesis, we analysed the drug sensitivity of four yeast strains with impaired protein degradation; Δ*ubc4*, Δ*ubc13*, Δ*doa4 *and Δ*qri8*. These deletion strains were not hypersensitive towards m-AMSA (or ICRF-187) (additional file 1 and table 2), suggesting that Δ*rad6 *cells are hypersensitive towards m-AMSA due to impaired PRR activity. The involvement of both HR repair and PRR in determining the sensitivity of yeast cells towards the topoisomerase II cleavage complex stabilising drugs mitoxantrone and idarubicin has previously been reported [23]. The observed lack of hypersensitivity of the Δ*rev1 *and Δ*rev3 *strains (additional file 1 and table 2) suggests that trans-lesion DNA synthesis plays no role in determining the sensitivity towards ICRF-187 or m-AMSA.

We also analysed the effect of deleting genes belonging to the nucleotide excision repair (NER) pathway – *RAD1 *and *RAD14*, the base excision repair (BER) pathway – *APN1*, and the mismatch repair (MMR) pathway – *MLH1*, *PMS1*, *MSH2 *and *MSH3*. None of these deletions caused cells to become more sensitive towards ICRF-187 and m-AMSA, indicating that these pathways are not involved in repairing DNA damage induced by these drugs. Interestingly, deleting genes involved in the MMR and NER pathways caused cells to become somewhat resistant to ICRF-187, and to a lesser extent towards m-AMSA (additional file 1 and table 2). The products of these genes have been implicated to have anti-recombination activities [24]. Increased levels of recombination in these cells could therefore be responsible for the observed low-level resistance towards ICRF-187.

### Deletion of DNA damage checkpoint genes has little effect on both ICRF-187 and m-AMSA sensitivity of yeast cells

While deleting genes involved in DNA repair caused cells to be hypersensitive towards both drugs tested, we observed little effect of deleting the DNA damage checkpoint genes *MEC3*, *DDC1*, *RAD17*, *TEL1*, *RAD9 *and *CHK1 *(additional file 1 and table 2). Our finding that checkpoint control regulation plays no important role for bisdioxopiperazine sensitivity supports earlier data showing that arresting yeast cells in G1 phase did not protect against ICRF-193 cytotoxicity [7]. The lack of importance of checkpoint function in determining sensitivity towards m-AMSA is also in accordance with published observations [23], where sensitivity towards the cleavage complex stabilising topoisomerase II poisons mitoxantrone, idarubicin, daunorubicin and doxorubicin were only marginally affected by inactivating the *RAD9*, *RAD17*, *MEC1*, and *RAD53 *genes, while the sensitivity of yeast cells towards the topoisomerase I poison camptothecin showed a strong dependency on these pathways.

### ICRF-187 is a possible substrate for the Pdr5 ABC transporter in yeast

In mammalian cells resistance towards various structurally-unrelated anti-neoplastic agents is often associated with over-expression of ABC-type drug efflux transporters such as p-glycoprotein and multi-drug resistance protein (MRP) as reviewed in [25]. Among the three yeast ABC transporters assessed in our study, Pdr5 is by far the best characterised [26]. While deleting *YOR1 *and *ATR1 *had no effect on drug sensitivity, Δ*pdr5 *cells were clearly hypersensitive towards ICRF-187 but not towards m-AMSA (additional file 1 and table 2), suggesting that ICRF-187 is a substrate for the Pdr5 pump in yeast. It has to be emphasized, that over-expression of drug efflux pumps has not been associated with resistance towards bisdioxopiperazines in mammalian cells.

### Transcriptional profiling of yeast cells after exposure to equitoxic concentrations of ICRF-187 and m-AMSA

In order to assess the effect on global gene expression of the interaction between human topoisomerase II α and the two drugs in yeast cells, transcriptional profiling was performed using Affymetrix gene chip technology. We exposed pMJ1-transformed JN362A_t2–4 _yeast cells expressing human topoisomerase II α as their sole active topoisomerase II to equitoxic doses of ICRF-187 and m-AMSA for two hours at 34°C. This treatment resulted in a 50 % reduction in clonogenic survival after exposure to both drugs (additional file 2). Genes whose average expression in two independent experiments was up- or down-regulated more than 1.5 fold by exposure to the drugs were filtered out. 138 transcripts were induced by exposure to ICRF-187 while the number was 90 for m-AMSA. 26 transcripts were repressed by exposure to ICRF-187 while the number was 16 for m-AMSA. Additional file 3 lists transcripts induced or repressed by ICRF-187 while additional file 4 lists transcripts induced or repressed by m-AMSA. The expression profile of selected genes is listed in Table 3 and discussed below.

**Table 3:** Selected genes induced or repressed by exposure of pMJ1-transformed yeast cells to equitoxic concentrations of ICRF-187 and m-AMSA for 2 hours

**Transcriptional activation**			
**Gene function**	**Gene Name**	**Effect of ICRF-187**	**Effect of m-AMSA**

**DNA damage**	*RNR3*	3.2	4.0
	*HUG1*	3.1	5.0
	*RAD51*	1.9	2.0
	*RAD54*	1.7	1.6
	*RNR2*	1.5	1.8
**Membrane transport**	*PDR12*	2.4	1.0
	*PDR15*	2.0	1.0
**Stress response**	*HSP12*	2.8	1.6
	*HSP26*	1.7	1.8
	*WSC4*	1.6	1.3
	*XBP1*	1.6	1.5
	*HSP42*	1.5	1.5
**Others**	*SWI1*	1.6	1.1
**Transcriptional repression**			
**Others**	*SNZ1*	0.6	1.1
	*PCL9*	0.7	0.6

### Genes induced by both drugs

Both compounds induced the expression of a number of genes known to be up-regulated by DNA damage. The expression of four well-established DNA-damage inducible genes; *RNR2*, *RNR3 *[27,28] and *RAD51*, *RAD54 *[29] was thus induced by both drugs. Both compounds also stimulated the expression of *HUG1 *recently shown to be up-regulated by DNA damage and replication arrest [30] (See Table 3, additional files 3 and 4). Furthermore, both drugs stimulated expression of the stress-inducible *XBP1 *gene whose protein product is a transcription factor. *XBP1 *expression is reportedly induced in response to heat shock, high osmolarity, oxidative stress, glucose starvation and DNA damage, and induces a slow-growth phenotype with lengthening of the G1 cell cycle phase [31]. The *PCL9 *gene product has cyclin-dependent protein kinase regulator activity suggesting a role for Pcl9p in cell cycle regulation [32]. Repression of *PCL9 *by exposure to both drugs (table 3, additional files 3 and 4) may thus be indicative of drug-induced cell cycle arrest in accordance with the *XBP1 *expression data. Finally, both drugs induced the expression of general stress-induced *HSP *genes as expected (table 3, additional files 3 and 4). Although expression of the *RNR3 *and *HUG1 *genes was up-regulated by both drugs, pMJ1-transformed cells having *RNR3 *or *SML1 *deleted (the latter is a functional non-inducible homolog of *HUG1*) have wild-type sensitivity towards both drugs (table 2, additional file 1), showing that although these genes are induced by both drugs, they are probably not involved in determining their cytotoxicity.

### Genes specifically induced by ICRF-187

We found that ICRF-187 specifically induced the expression of two genes encoding the ABC efflux transporters Pdr12 and Pdr15, while m-AMSA had no effect on the expression of these genes (table 3, additional files 3 and 4). Transcription of the stress-inducible *WSC4 *gene was likewise enhanced by exposure to ICRF-187 (table 3, additional files 3 and 4). Knocking out *WSC4 *in yeast cells has been found to enhance their sensitivity towards various stresses including heat, ethanol and DNA damage [33]. Recently, the SWI/SNF complex was directly shown to repress transcription in *S. cerevisiae *cells [34]. We found that *SWI1 *was specifically induced by ICRF-187 (table 3, additional files 3 and 4). Finally, we found that ICRF-187 specifically repressed the expression of the stationary phase-induced *SNZ1 *gene [35] (table 3, additional files 3 and 4).

### Exposure of yeast cells to ICRF-187 causes less transcriptional induction of DNA damage-inducible genes than exposure to m-AMSA at equitoxic drug concentrations

**Figure 2:**
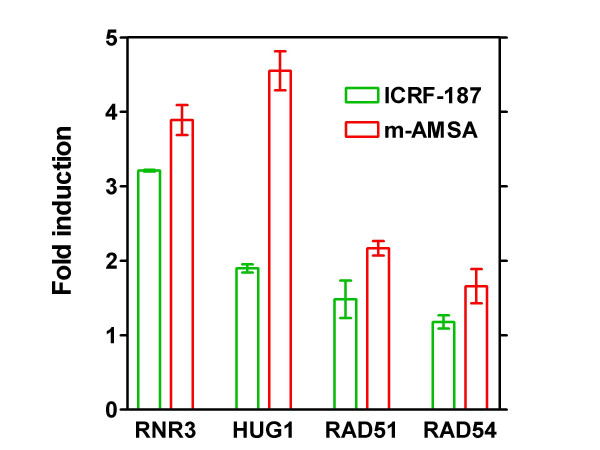
Analysis of gene expression by real time PCR. Real-time PCR was used to determine the expression of the DNA-damage inducible genes *RNR3*, *HUG1*, *RAD51*, and *RAD54 *by using the 2-^ΔΔCt ^method. Gene expression was normalized to that of the actin gene *ACT1*. It can be seen that exposure of yeast cells to m-AMSA results in higher levels of induction of transcription of these four genes than exposure to ICRF-187 when the two drugs are applied at equitoxic concentrations. Error-bars represent SEM of two independent experiments each performed in duplicate.

To verify the array data we performed real-time PCR to assess the expression of the *RNR3*, *HUG1*, *RAD51 *and *RAD54 *genes after exposure to the two drugs using the actin gene *ACT1 *as internal control (figure 2). Real-time PCR confirmed induction of these established DNA damage-inducible genes by both drugs assessed. Furthermore, exposure of the cells to m-AMSA resulted in a higher level of induction than did exposure to ICRF-187 for the four genes tested, especially for *HUG1*. These data suggest that when yeast cells are exposed to equitoxic concentrations of the two drugs, m-AMSA generates more extensive DNA damage than ICRF-187.

### ICRF-187 sensitivity of mammalian hamster cells depends on their homologous recombination status, albeit to a lesser extent than seen for m-AMSA sensitivity

The yeast clonogenic assays presented above point to an important role of HR in the repair of m-AMSA-induced DNA damage, while the importance of this pathway in the repair of ICRF-187-induced DNA damage is less so. Because HR is the major repair pathway in yeast [21], while both NHEJ and HR are important for the repair of DNA breaks in mammalian cells [36], we next turned to assess the importance of these pathways in mammalian cells having reduced levels of HR and NHEJ. In this analysis we used a panel of four hamster cell lines; AA8 cells (wild-type), irs1SF cells [37] (recombination defective caused by non-functional XRCC3), CXR3 cells [37] (recombination proficient due to ectopic expression of human XRCC3), and V3-3 cells [38] (reduced level of NHEJ due to non-functional DNA-PK_cs_).

**Figure 3:**
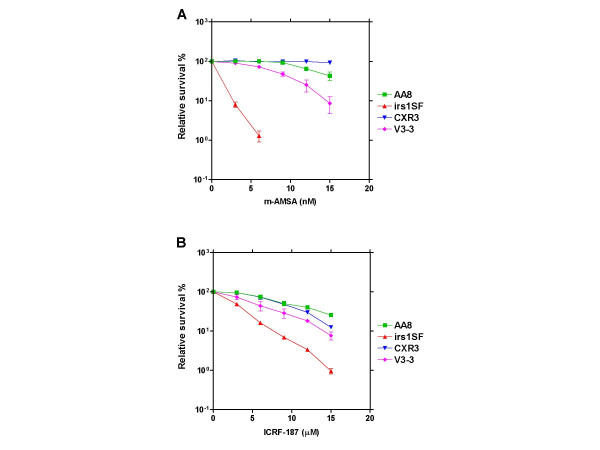
Assessing the clonogenic sensitivity of HR and NHEJ deficient and proficient hamster cells towards ICRF-187 and m-AMSA. To determine the sensitivity of the four cell lines AA8 (wild-type), irs1SF (recombination defective caused by non-functional XRCC3), CXR3 (recombination proficient due to ectopic expression of human XRCC3), and V3-3 (defective in NHEJ due to non-functional DNA-PK_cs_) towards ICRF-187 and m-AMSA, a clonogenic assay with continuous drug exposure was used. Error-bars represent SEM of two independent experiments.

We observed a strong dependence on HR for the sensitivity towards m-AMSA (figure 3A). Thus, only 1 % relative survival was seen for the irs1SF cells (recombination defective) at 6 nM of this drug, while wild-type AA8 cells were only slightly sensitive to15 nM m-AMSA. Furthermore, ectopic expression of human *XRCC3 *fully reversed the m-AMSA hypersensitivity as CXR3 cells were no more hypersensitive than AA8 wild-type cells, confirming the notion that HR plays a role in the repair of topoisomerase II-induced DNA breaks in mammalian cells. We also observed that irs1SF cells were hypersensitive towards ICRF-187 (figure 3B), but the degree of hypersensitivity was much less than observed for m-AMSA, as also seen for recombination deficient yeast cells. Again, ectopic expression of the human *XRCC3 *homolog reversed the hypersensitivity as CXR3 cells displayed near wild-type sensitivity towards ICRF-187.

### DNA-PK_cs _deficient hamster cells show slightly increased sensitivity towards both m-AMSA and ICRF-187

To assess the effect of NHEJ on drug sensitivity we also employed the V3-3 cell line (DNA-PK_cs _deficient, with concomitant reduced level of NHEJ). The results of these experiments are depicted in figure 3A and 3B. The V3-3 cells were slightly hypersensitive towards both drugs suggesting a role for NHEJ in the repair of DNA lesions induced by both drugs.

### AA8, irs1SF, CXR3 and V3-3 cells have similar levels of topoisomerase II catalytic activity

The sensitivity of cells towards topoisomerase II directed drugs depends both on their levels of topoisomerase II catalytic activity, and on their capability to repair topoisomerase II-induced DNA damage. We therefore determined the level of topoisomerase II catalytic (DNA strand passage) activity in crude protein extracts isolated from the four cell lines used in clonogenic assays, by applying a radioactive decatenation assay. No significant difference in the level of topoisomerase II DNA strand passage activity was recorded between the four cell lines (additional file 5). This result rules out the possibility that varying levels of topoisomerase II catalytic activity in these cells is responsible for their differential drug sensitivity.

### ICRF-187 induces lower levels of homologous recombination in hamster cells than m-AMSA at equitoxic concentrations

**Figure 4:**
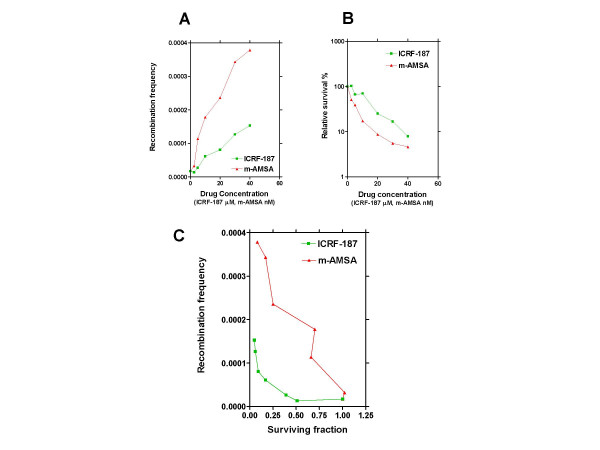
Assessing the effect of equitoxic concentrations of ICRF-187 and m-AMSA on the level of HR in SPD8 hamster cells. The SPD8 cell line has a defective *hprt *gene that can be repaired by HR. Panel A depicts the induction of HR induced by increasing concentrations of the two drugs. Panel B depicts the relative survival of cells exposed to similar concentrations of the two drugs. The data represented in Panel A and B was used to generate Panel C, where recombination frequency is plotted against the surviving fraction of cells. This data presentation allows a direct comparison of recombination levels at equitoxic concentrations of the two drugs. Representative data from one of three independent experiments is shown.

The hypersensitivity of the recombination defective irs1SF cells towards both drugs suggests that HR is involved in repairing DNA lesions induced by both drugs. To address this directly we applied a mammalian recombination assay to measure stimulation of HR by ICRF-187 and m-AMSA by using SPD8 hamster cells [39]. This assay measures the repair of a defective chromosomal *hprt *gene by the activity of HR. From figure 4A it is evident that both drugs stimulated the level of HR in a dose dependent manner. When recombination frequency is expressed as a function of surviving cells (figure 4C) it becomes evident that the recombination frequency increases with increasing cell mortality for both drugs tested. From figure 4C it is also evident that at equitoxic concentrations of the two drugs, m-AMSA stimulated HR to much higher levels than did ICRF-187. Thus, at 50 % survival, no induction of HR was seen with ICRF-187 (in three independent experiments), while m-AMSA caused an approximately 10-fold induction at equitoxic doses.

### ICRF-187 induces only low levels of H2AX phosphorylation in human SCLC cells as compared to m-AMSA

**Figure 5:**
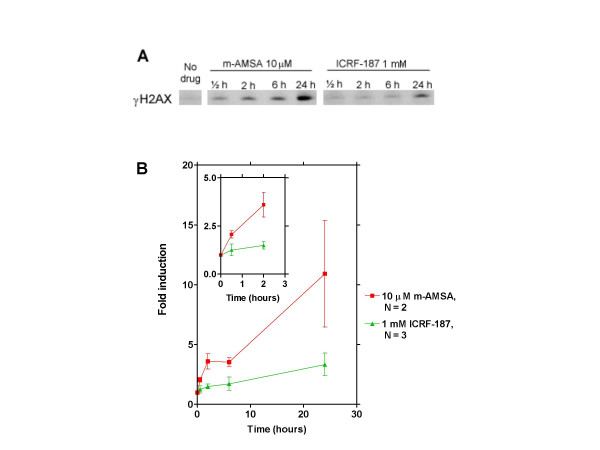
Assessing the effect of m-AMSA and ICRF-187 on γH2AX induction in human SCLC OC-NYH cells. To assess the level of DNA breaks in human cells after exposure to 10 μM m-AMSA and 1 mM ICRF-187 for increasing time points, total histones were isolated after incubation with the drugs. 10 μg of purified histones was then used in western blotting experiments. Panel A depicts a typical Western blot showing increased γH2AX induction with increasing drug incubation times. Panel B depicts fold γH2AX induction plotted against drug incubation time to analyse the kinetics of induction of DNA double strand breaks by the two drugs. Insert shows γH2AX induction from 0 to 2 hours for better resolution. Error-bars represent SEM of three independent experiments for ICRF-187 treatment and SEM of two independent experiments for m-AMSA treatment.

Induction of γH2AX is a well-established marker for topoisomerase-induced DNA double strand breaks in mammalian cells [40-42]. We therefore assessed the effect of exposing human SCLC OC-NYH cells to 10 μM m-AMSA and 1 mM of ICRF-187 at increasing time points (figure 5A and 5B). Exposure to 10 μM m-AMSA quickly resulted in γH2AX induction. Thus, induction was evident after 30 min, and after 24 hours more than 10-fold induction was observed. In contrast, when cells were exposed to 1 mM ICRF-187, much less γH2AX induction was observed, and after 24 hours the level of induction was less that three-fold.

## Discussion

We initiated the study by assessing the clonogenic sensitivity of yeast single-gene deletion mutants ectopically expressing human topoisomerase II α towards m-AMSA and ICRF-187. The results presented in table 2 and additional file 1 indicates that HR plays a role in the repair of ICRF-187-induced DNA damage. Previous studies addressing the bisdioxopiperazine sensitivity of yeast cells have generated different results. In one study *rad52*-cells had the same sensitivity towards ICRF-187 and ICRF-193 as did *RAD52*+ cells [7], while in other studies, HR deficient cells were found to be hypersensitive towards bisdioxopiperazines, although to a much lesser extent than towards topoisomerase II cleavage complex stabilising drugs [43,44]. Our present study involving numerous other genes involved in various aspects of HR clearly establishes this pathway as being a functional determinant for bisdioxopiperazine sensitivity in yeast cells. In a recent work by Simon and colleagues, where a panel of yeast deletion strains was also applied to pinpoint the mechanism of action of various anticancer drugs, a given drug was classified as selective if one single pathway was mainly involved in determining cellular sensitivity [23]. The selective involvement of the HR pathway in determining the sensitivity towards ICRF-187 classifies this drug as highly selective according to this definition. However, it is important to note that although HR clearly does play a role in protecting yeast cells from ICRF-187 cytotoxicity, the importance of this pathway on cell survival in the presence of m-AMSA is much greater (additional file 1, table 2) – in accordance with this drug being a topoisomerase II poison killing cells solely by the generation of topoisomerase II-mediated DNA breaks.

We find that the relative sensitivity of AA8, irs1SF, and CXR3 cells towards m-AMSA (figure 3) closely resembles their sensitivity towards etoposide [45], showing that the hypersensitivity of *XRCC3 *deficient irs1SF cells is general to topoisomerase II poisons, suggesting a role for HR in the repair of topoisomerase II-induced DNA breaks in these cells. The involvement of HR in the repair of DNA lesions induced by topoisomerase II poisons in higher eukaryotes is also supported by a recent work suggesting that RAD51 plays an important role in the repair of etoposide-induced DNA damage in human small cell lung cancer cells [46], and by work by Adachi and colleagues who recently found that knocking out *RAD54 *in chicken DT40 cells enhances their sensitivity towards the topoisomerase II poison etoposide [13].

We find that XRCC3 defective irs1SF cells are more sensitive towards ICRF-187 than the parental AA8 cells, although the XRCC3 defect has a much more pronounced effect on m-AMSA sensitivity (figure 3A versus figure 3B) – as seen with the yeast deletion mutant panel. Adachi and colleagues have found that knocking out *RAD54 *in DT40 chicken cells does not increase sensitivity towards ICRF-193 [13]. The reason for this discrepancy in not clear. The difference observed between the DT40 and irs1SFcells may relate to the fact that different DNA repair genes are deleted in the two cell lines possibly resulting in different processing of bisdioxopiperazine-induced DNA damage. In any case, this discrepancy does not challenge the overall finding that HR plays a more important role in protecting cells of various origin from cytotoxicity induced by topoisomerase II poisons as compared to cytotoxicity induced by bisdioxopiperazines.

To study the importance of NHEJ in determining the sensitivity towards m-AMSA and ICRF-187 we employed a DNA-PK_cs _defective hamster cell line, V3-3, which has reduced levels of NHEJ activity. We find that V3-3 cells are hypersensitive towards m-AMSA (figure 3A). This result is in accordance with a recent publication by Willmore and colleagues who found that a specific small-molecule inhibitor of DNA-PK_cs _NU7026 could potentate the sensitivity of human leukemic K562 cells towards various topoisomerase II poisons [47]. Our result is also in accordance with a recent report by Adachi and colleagues showing that DNA-PK_cs _knockout chicken DT40 cells are hypersensitive towards etoposide [48]. These result points towards an important role of DNA-PK_cs _in determining the sensitivity of higher vertebrate cells towards topoisomerase II poisons. Different studies have demonstrated a more pronounced effect of inactivating Ku as compared to DNA-PK_cs _on cellular sensitivity towards topoisomerase II poisons [48-50]. Consequently, the importance of NHEJ in determining the sensitivity towards topoisomerase II poisons in mammalian cells is likely to be underestimated from our V3-3 cell data. This notion is confirmed by early publications demonstrating that Ku deficient hamster cells are highly sensitive towards m-AMSA and etoposide [51,52].

Our finding that V3-3 cells are more sensitive towards ICRF-187 than AA8 cells (figure 3B) is also in accordance with observations by Adachi and colleagues who find that DNA-PK_cs _deficient chicken DT40 cells are hypersensitive towards another bisdioxopiperazine analog, ICRF-193. These authors found the effect of inactivating DNA-PK_cs _to be much more pronounced than seen in our present study. While the reason for this difference is not clear, it has to be mentioned that studies addressing the effect of DNA-PK_cs _on the sensitivity towards topoisomerase II targeting drugs and ionising radiation have produced varying results. Thus, in a study by Jin and colleagues, DNA-PK_cs _defective murine cells were much less sensitive towards etoposide than Ku70 and Ku80 deficient cells [50], while in a study by Gao and colleagues the importance of DNA-PK_cs _on the sensitivity towards ionising radiation was found to depend on cell type and/or cell cycle distribution [49]. Such variation could well explain the different importance of DNA-PK_cs _observed in our study and in the work by Adachi and colleagues.

In order to study directly the effect of exposing mammalian cells to ICRF-187 and m-AMSA on the levels of HR, we employed a mammalian recombination assay previously described [39]. In this assay, m-AMSA enhanced the level of recombination in SPD8 cells to higher levels than ICRF-187 at all cytotoxicity levels tested (figure 4C), demonstrating directly pronounced differences in the mechanism(s) by which topoisomerase II poisons and bisdioxopiperazines kill cells. This notion is further confirmed by our γH2AX induction experiments, where ICRF-187 causes much lower levels of induction (figure 5), demonstrating that ICRF-187 induces less DNA breaks in cells than m-AMSA. Our observation that ICRF-187 induces both HR and γH2AX induction in mammalian cells, is in agreement with a recent paper demonstrating by the use of comet assay and pulsed field gel electroforesis that ICRF-193 induces DNA breaks in mammalian cells [16]. This result is also in agreement with our real-time PCR results where ICRF-187 tended to induce the expression of established DNA damage-inducible genes.

The finding that ICRF-187 induces lower levels of HR than m-AMSA in SPD8 cells at equitoxic doses may be explained in at least two ways. Bisdioxopiperazine-induced DNA breaks could be more toxic to cells than breaks induced by topoisomerase II poisons, or the DNA breaks could be only partly responsible for killing the cells. Three lines of evidence support the latter possibility. (*i*) Functional ATR, but not ATM, is required for a cell cycle checkpoint arrest induced by ICRF-193 [53], suggesting that DNA breaks are not involved in triggering the checkpoint signal. (*ii*) Exposure of mammalian cells to the topoisomerase II poison etoposide induces degradation of the large subunit of RNA polymerase II indicative of DNA breaks, while this is not the case for ICRF-193 [14]. (*iii*) Our finding that cell survival in the presence of ICRF-187 depends less on HR than cell survival in the presence of m-AMSA suggests that ICRF-187-induced DNA breaks contribute less to overall cytotoxicity than m-AMSA-induced DNA breaks. If ICRF-187-induced DNA breaks were more toxic to cells than m-AMSA-induced DNA breaks, cell survival in the presence of ICRF-187 would be expected to depend at least as much on HR as cell survival in the presence of m-AMSA. This is not the case.

What mechanisms are then responsible for producing the DNA breaks induced by bisdioxopiperazines in cells? In a recent work by Oestergaard and colleagues).)[17], it is suggested that the toxic intermediate causing bisdioxopiperazine cytotoxicity is topoisomerase II stably bound to two DNA segments – a conformation they suggested would only be attainable if the DNA strand passage reaction of topoisomerase II is functioning. HR could then be required for the repair of DNA breaks generated by the collision of DNA tracking complexes with such four-way DNA junctions / topoisomerase II closed clamp complexes on DNA. It has recently been demonstrated by the use of pulsed field gel electrophoresis, that inhibiting DNA replication by aphidicolin does not reduce the level of DNA breaks generated by exposure of mammalian cells to ICRF193, while the level of m-AMSA-induced DNA breaks was reduced by aphidicolin treatment [16]. This result suggests that collision of the DNA replication complex with bisdioxopiperazine-induced topoisomerase II closed clamp complex on DNA is not involved in generating the DNA breaks.

In a recent study by Lundin and colleagues, it was demonstrated that inhibiting DNA replication by exposing cells to hydroxyurea resulted in the generation of DNA breaks [54]. Furthermore, in this work as well as in a subsequent work [45], HR was shown to be functionally involved in repairing such DNA breaks. The first of these two studies used the same four hamster cell lines that are also used in our present study. Remarkably, the relative sensitivity of these cell lines towards hydroxyurea exactly resembles their sensitivity towards ICRF-187 seen in our present work. This may suggest that replication arrest is involved in generating DNA breaks induced by bisdioxopiperazines in cells. Here, replication forks stalled at the bisdioxopiperazine-induced closed clamp complexes could be the source of DNA breaks in newly replicated DNA [54]. This would also explain the lack of effect of aphidicolin on the level of ICRF-193-induced DNA breaks observed by Hajji and colleagues [16]. If the DNA breaks result from arrested replications forks, and not from the collision of the DNA replication complex with the closed clamp complex on DNA, no effect of aphidicolin would be expected. This mechanism would also explain why yeast cells arrested in intra-S phase are not protected from ICRF-193 cytotoxicity [7]. We therefore suggest that this mechanism is responsible for generating DNA breaks induced by bisdioxopiperazines in cells.

Together our HR, γH2AX, and cytotoxicity data suggest that bisdioxopiperazines kill cells by a combination of DNA break-related and DNA break-unrelated mechanisms. This raises the question as to which mechanism(s) is / are involved in mediating the DNA break-unrelated part of bisdioxopiperazine cytotoxicity. Exposure of mammalian cells to ICRF-193 represses global transcription and mediates selective degradation of topoisomerase II β via a transcription dependent mechanism [14]. Inhibition of the RNA polymerase II – transcription complex by bisdioxopiperazine-induced topoisomerase II complexes on DNA could therefore be involved in mediating the DNA break-unrelated component of bisdioxopiperazine cytotoxicity.

Treatment of mammalian cells with high doses of ICRF-187 for one hour is capable of antagonising DNA breaks and the cytotoxicity of topoisomerase II poisons [55,56], and this antagonism can be extended to animal models, where ICRF-187 can antagonise etoposide toxicity [57,58] and bone marrow depression (unpublished results). How are bisdioxopiperazines capable of antagonising the effects of topoisomerase II while at the same time producing DNA breaks? Two independent studies assessing the dose- and schedule-dependency of combinations of bisdioxopiperazines and topoisomerase II poisons on cytotoxicity in mammalian cells may provide important clues. One study investigated the effect of combinations of ICRF-193 and etoposide [59]. Here, continuous administration of low doses of both drugs resulted in synergistic cell kill, while treatment with high concentrations of ICRF-193 for one hour efficiently antagonised etoposide-mediated cytotoxicity. A similar effect of schedule and concentration on cytotoxicity has also been observed for combinations of ICRF-187 and daunorubicin [60], but here long time exposure of the cells to both drugs resulted in an additive effect on cell kill. We have previously shown that exposure of mammalian cells to high concentrations of ICRF-187 (500 – 1000 μM) alone for 60 min is non-toxic, and that this treatment efficiently antagonises etoposide-induced DNA breaks and cytotoxicity [61,62]. In these studies, exposure of cells to 200 μM ICRF-187 was found to trap most cellular topoisomerase II α and β as non-extractable complexes on DNA. The inability of topoisomerase II poisons to act on bisdioxopiperazine-stabilised closed clamp complexes on DNA could therefore explain the antagonistic effect of high concentrations of bisdioxopiperazines generally observed in one-hour drug exposure experiments [59-62]. When a low concentration of bisdioxopiperazine is administered, it is most likely that only a small fraction of the topoisomerase II molecules in the cell is trapped as closed clamp complexes on DNA, leaving some or most topoisomerase II molecules available for the action of topoisomerase II poisons. Therefore, after long-time exposure of cells to low concentrations of bisdioxopiperazine and a topoisomerase II poison, covalent and non-covalent complexes of topoisomerase II on DNA could both contribute to cytotoxicity by generating DNA breaks via different mechanisms, thus explaining the additive or synergistic effect on cell kill observed under these circumstances.

To summarise, our data are consistent with a model where bisdioxopiperazine-induced cytotoxicity results from a combination of DNA break-related and -unrelated mechanisms, where the DNA-break unrelated mechanism is clearly not mediated by the inhibition of catalytic topoisomerase II activity in the cells.

## Conclusion

Since the discovery by Andoh and colleagues in 1991, that the bisdioxopiperazines target eukaryotic topoisomerase II [63,64], their mode of cytotoxicity has been the cause of debate. While early publications tended to classify these compounds as "pure" catalytic inhibitors of topoisomerase II, expected to kill cells by depriving them of essential topoisomerase II catalytic activity, numerous recent reports present data that are not consistent with this view [7,11-14,16,17]. In the present report we have characterised bisdioxopiperazine (ICRF-187) induced cytotoxicity in yeast and mammalian cells by using a combination of genetic and molecular approaches. Our results are consistent with a model where bisdioxopiperazines cause cytotoxicity by stabilising a topoisomerase II reaction intermediate / complex on DNA inducing DNA breaks in cells which are repaired by HR and NHEJ. We propose that cells exposed to bisdioxopiperazines die by a combination of DNA break-related and-DNA break-unrelated mechanisms. Our study clearly establishes that bisdioxopiperazines do not kill cells solely by depriving them of topoisomerase II catalytic activity.

## Methods

### Drugs

ICRF-187 (Cardioxane, Chiron group) was dissolved in sterile water at 20 mg/ml and kept at – 80°C. To avoid hydrolysis of the drug, fresh aliquots were used for each experiment. m-AMSA (Pfizer) was diluted in DMSO and stored at – 80°C at 1 mg/ml. L-azaserine and thymidine (both from Sigma) were added directly to tissue culture medium. 6-thioguanine and hypoxanthine (both from Sigma) were dissolved in 5 M NaOH and immediately added to the tissue culture medium.

### Yeast strains and constructs

BY4741 haploid *Saccharomyces cerevisiae *cells (*MAT***a ***his3*Δ*1 leu2*Δ*0 met15*Δ*0 ura3*Δ*0*) and a panel of single-gene deletion derivatives hereof (table 1) were purchased from EUROSCARF, Institute of Microbiology, Johann Wolfgang Goethe University Frankfurt, Germany. The construction of BY4741 and its deletion derivatives have been described [65]. JN362A_t2–4 _cells with the relevant genotype (*MAT***a ***ura3*–*52 leu2 trp1 his7 ade1*–*2 ISE2 top2*-*4*) were kindly provided by Dr. John L. Nitiss, St. Jude Children's Research Hospital, Memphis TN, USA. This strain and the construct for functional expression of human topoisomerase II α in yeast pMJ1 (*URA3*) have been described previously [66]. All yeast strains were transformed with pMJ1 to functionally express human topoisomerase II α in a cell cycle independent fashion. BY4741 wild-type and Δ*rad6*, Δ*rad50*, Δ*rad52*, Δ*sae2 *and Δ*yku70 *cells were also transformed with an empty *URA3 *vector (pYX112). The ICRF-187 and m-AMSA sensitivity of pYX112-transformed cells was assessed to assure that the drug sensitivity of the pMJ1-transformed cells (Table 2, S1) is related to the ectopic expression of human topoisomerase II α in the cells, which was the case. Transformation and selection was carried out according to standard procedures using lithium acetate cell wall permeabilisation and PEG-mediated DNA uptake by using single-stranded DNA as carrier as described [67]. Selection was done on SC-URA plates. Three independent pMJ1-transformed yeast clones were selected and propagated for each transformation. All strains were propagated at 30°C, to be subsequently used in clonogenic assays at 34°C.

### Yeast clonogenic assay

The clonogenic sensitivity of the yeast cells towards ICRF-187 and m-AMSA was determined using a clonogenic assay essentially performed as described in [7]. Briefly, overnight cultures of the strains were grown in SC-URA medium at 34°C at 200 rpm. Cells in log phase were diluted to 2 × 10^6 ^cells/ml in pre-warmed YPD medium, and 3 ml cultures were exposed to different concentrations of drug at 34°C for 22.5 hours. After drug exposure the samples were diluted up to 10^5 ^times (depending on the combination of strain and drug used) in distilled sterile water. Yeast cells that were not diluted before plating were spun down by brief centrifugation, and re-suspended in the same volume of sterile water. Next, 200 μl of diluted cells were transferred to SC-URA plates, which were incubated for 5 days at 30°C before counting. 200 to 600 colonies were typically counted for each drug concentration in each single experiment. Finally, the relative survival at the different drug concentrations as compared to the no drug sample was calculated to generate dose-response curves. For each combination of yeast strain and drug, at least three dose-response curves were generated using pMJ1-transformed cells from at least two independent clones (mostly from three).

### Yeast microarray gene expression analysis

Microarray experiments were performed with yeast strain JN362_t2–4 _transformed with pMJ1 to functionally express human topoisomerase II α. Fresh colonies were inoculated into YPD medium and grown overnight at 34°C, 180 rpm. The cultures were then diluted to obtain an OD_600 _of 0.2. Cultures of 50 ml in YPD medium were first grown for two hours to assure exponential growth of the cells. 1 mg/ml ICRF-187 or 50 μg/ml m-AMSA (equitoxic concentrations) were then added to the cell cultures (a no-drug sample was also included), and the cells were grown for an additional two hours. Each treatment was performed in duplicate. The used concentration of both drugs resulted in a reduction in the clonogenecity of the cells of 50 %. After treatment cells were harvested by centrifugation. Total RNA was isolated by the hot acidic phenol method [69]. All the steps for cDNA synthesis, cRNA synthesis, biotin labeling and array hybridization to Affymetrix S98 yeast arrays were performed as described in the Affymetrix GeneChip Expression Analysis Technical Manual (Affymetrix), and performed at the microarray core facility at Rigshospitalet, Copenhagen Denmark. Briefly, cDNA was synthesized from 5 μg RNA using a (dT)_24 _primer containing a T7 RNA polymerase promoter sequence and SuperScript II reverse transcriptase (Invitrogen) for 1 h at 42°C followed by second-strand synthesis using DNA polymerase I and RNase H digestion followed by isolation of cDNA using GeneChip Sample Cleanup Module (Affymetrix). The cDNA was used as template for synthesis of biotin-labeled cRNA by incubation with biotin-labeled ribonucleotides and T7 RNA polymerase for 5 h at 37°C. Biotin-labeled cRNA was purified using GeneChip Sample Cleanup Module. Biotinylated cRNA was fragmented and 15 μg used for hybridization to Affymetrix Yeast Genome S98 arrays at 45°C for 16 h as described in the Affymetrix users' manual. Washing and array staining with streptavidin-phytoerythrin were performed using the GeneChip Fluidics Station 400 and scanning was performed with a Gene Array Scanner G25 (Agilent technology). Data was analyzed using the DNA-Chip Analyzer (dChip) software [70].

### Real-time PCR analysis

The RNA preparations used for microarray analyses were also used for real-time PCR. This analysis was performed on an ABIPrism 7900HT (Applied Biosystems). RNA samples were DNase treated using the DNA-free™ DNase treatment and removal kit (Ambion), and RNA concentrations were measured before conversion to cDNA using the TaqMan RT kit (Applied Bioscience). Priming was performed by random hexamers converting 2 μg RNA pr 100 μl reaction volume, to make 20 ng/μl cDNA. Primers were designed for coding sequences from the Saccharomyces genome database  using the Primer 3 input program . All primers were purchased at DNA Technology A/S, with melting temperatures close to 60°C. Reaction mixtures containing the following components at the indicated end-concentrations were prepared. To make a total of 40 μl in sterile water, 20 μl 1x SYBR^® ^green PCR master mix (Applied Biosystems), 250 nM forward primer, 250 nM reverse primer, and 5 ng template was mixed. Cycling conditions: 95°C for 10 min, followed by 40–45 cycles of 95°C for 15 s and 60°C for 60 s. Relative values of gene expression were calculated with untreated samples as calibrator, and normalized to levels of actin, according to the 2^-ΔΔCt ^method [71] and (User bulletin #2, AbiPrism 7700 Sequence Detection System, Applied Biosystems) after primer optimisation and target efficiency evaluation. The following primers were used:

*HUG1*-forward, AGGCCTTAACCCAAAGCAAT;

*HUG1*-reverse, TCTTGTTGACACGGTTGCTC;

*RNR3*-forvard, ATGCATCTCCAGTTCCATCC;

*RNR3*-reverse, GGGGCAACACTATCTTCCAA;

*RAD51*-forward, GTGGCGGTGAAGGTAAGTGT;

*RAD51*-reverse, GTCTAATCCGAACCGCTGAG;

*RAD54*-forward, CTAAAGCAGGTGGGTGTGGT;

*RAD54*-reverse, CTTGTTGATCAGCAGCAGGA;

*ACT1*-forward, CGGTGATGGTGTTACTCACG;

*ACT1*-reverse, GGCCAAATCGATTCTCAAAA.

### Mammalian cells

The CHO cell lines AA8, irs1SF, CXR3, V3-3, and the hamster lung fibroblast cell line SPD8 were kindly provided by Dr Thomas Helleday, University of Sheffield, UK. AA8 is a wild-type cell line. The AA8-derived irs1SF cell line is XRCC3-defective and has reduced levels of HR [37]. CXR3 is a human-*XRCC3*-cosmid complemented strain of irs1SF, which is proficient in HR [37]. The V3-3 cell line is DNA-PK_cs_-deficient and consequently deficient in NHEJ [38]. The SPD8 cells carry a non-functional *hprt *gene that can be repaired by HR [39]. HPRT^+ ^cells can then be selected on HAsT medium containing hypoxanthine, L-azaserine and thymidine. When SPD8 cells were not used in the recombination assay they were propagated in medium supplemented with 6-thioguanine to select against spontaneous reversion to the HPRT^+ ^phenotype. Human SCLC OC-NYH cells have been described [72]. Hamster cells were propagated in DMEM medium and OC-NYH cells were propagated in RPMI-1640 medium. All cell culture media were supplemented with 10 % fetal calf serum and 100 U/ml penicillin-streptomycin. Cells were grown in a humidified atmosphere containing 5 % CO_2 _in the dark at 37°C.

### Determination of topoisomerase II activity in crude cell extracts

Topoisomerase II activity in crude extract was determined by using a decatenation assay previously described [68]. Briefly, 200 ng ^3^H labeled kDNA isolated from *C. fasciculata *was incubated with increasing amounts of crude extracts in 20 μl reaction buffer containing 10 mM TRIS-HCl pH 7.7, 50 mM NaCl, 50 mM KCl, 5 mM MgCl_2_, 1 mM EDTA, 15 μg/ml BSA and 1 mM ATP for 20 min at 37°C. After addition of 5x stop buffer (5 % Sarkosyl, 0.0025 % bromophenol blue and 50 % glycerol), unprocessed kDNA network and decatenated DNA circles were separated by filtering, and the amount of unprocessed kDNA in each reaction was determined by scintillation counting. The amount of crude extract required to fully decatenate 200 ng of kDNA under these assay conditions (which is equivalent to 1 U of catalytic activity) was then determined, and the specific activity of the crude extract was calculated as U/μg protein.

### Mammalian clonogenic assay

Four hours prior to continuous treatment with either ICRF-187 or m-AMSA, 250 cells of each of the hamster cell lines were plated onto 100 mm dishes. After 7 days colonies were fixed and strained in methylene blue in methanol (4 mg/ml), and colonies with more than 50 cells were counted. Finally, the relative survival compared to the no drug treatment was calculated, and plotted against drug concentrations to generate dose-response curves. 150 – 250 colonies were typically counted in the "no drug" dishes.

### Mammalian homologous recombination assay

A mammalian recombination assay was performed as described [39]. Briefly, 1 × 10^6 ^SPD8 cells were inoculated into 75 cm^2 ^flasks. When transferred, 6-thioguanine was omitted from the medium. Cells were trypsinised and resuspended in 10 ml medium at 100,000 cells/ml, and exposed to the indicated drug concentrations for 24 hours. To determine clonogenic survival, for each drug-treatment 500 cells were transferred to each of two 100 mm petri dishes containing 10 ml of non-selecting medium and the cells were cultured for 7 days. For selection of recombination events, 300,000 cells were transferred to each of three 100 mm petri dishes containing 10 ml medium supplemented with 50 μM hypoxanthine, 10 μM L-azaserine and 5 μM thymidine and selection was carried out for 10 days. Colonies were fixed by using methylene blue in methanol (4 mg/ml) and counted. Finally, the recombination frequency was determined as the plating efficiency in recombination selective medium divided by the plating efficiency in normal medium, for all concentrations of ICRF-187 or m-AMSA. To enable comparison of recombination frequency at equitoxic levels of m-AMSA and ICRF-187, the recombination frequency was plotted against the relative clonogenic survival of cells receiving only drug.

### Histone purification

Human SCLC OC-NYH cells were grown to sub-confluence and histones were extracted as follows. After the relevant drug treatments, the cells were pelleted and washed in cold PBS, and lysed in lysis buffer (10 mM TRIS-HCl pH = 6.5, 50 mM Sodium Bisulphate, 1% Triton X-100, 10 mM MgCl, 8.6% sucrose) at 4°C by applying 20 strokes in a tight fitting Dounce homogenizer. Released nuclei were pelleted by centrifugation at 2500 g for 10 min at 4°C, and washed in lysis buffer followed by wash buffer (10 mM TRIS-HCl, 13 mM EDTA pH 7.4). The pellet was next resuspended in 100 μl ice-cold 0.4 M H_2_SO_4_, and incubated for 1 hour at 4°C prior to centrifugation. The supernatant was transferred to a clean tube and 1 ml ice-cold acetone was added followed by incubation overnight for histone precipitation. After centrifugation, the pellet was air-dried and resuspended in 40 μl H_2_O, and the protein concentration was determined by Bradford protein assay (Bio-Rad Laboratories)

### γH2AX western blot

Western blotting was performed by loading 10 μg of total histones on a 4–12% gradient gel (NuPageTM Bis-Tris Gel, Invitrogen). Separated proteins were transferred to nitrocellulose membranes (Bio-Rad) which were blocked in 10% skimmed milk (Fluka) and incubated overnight with anti γH2AX primary antibody diluted 1:500 (Upstate Technology, cat no 16–193) followed by detection with goat-anti-mouse (Amersham) 1:2000 for one hour. Detection with ECL Plus™(Amersham) was performed by scanning on STORM™ 840 (Molecular Dynamics Inc), on which the image was optimized and bands quantified by Image Quant™ version 5.0 (Molecular Dynamics).

## List of abbreviations used

BER, Base Excision Repair; CHO, chinese hamster ovary; HR, Homologous Recombination; ICRF-187, (+)-1,2-bis(3,5-dioxopiperazinyl-1-yl)propane; m-AMSA, (N-[4-(9-acridinylamino)-3-methoxyphenyl]methanesulphonanilide); MMR, Mismatch Repair; NER, Nucleotide Excision Repair; NHEJ, Non-Homologous End Joining; PCR, Polymerase Chain Reaction; PRR, Post Replication Repair; SCLC, Small Cell Lung Cancer; SC-URA, Synthetic medium lacking uracil; SSA, Single Strand Anealing; YPD, Medium containing Yeast extract, Peptone and Dextrose.

## Authors' contributions

**Lars H. Jensen: **Participated in planning the experiments, performed yeast transformations and clonogenic assays, and prepared the manuscript. **Marielle Dejligbjerg**: Performed γH2AX western blots, primer design, real-time PCR experiments, and data quantitation. **Lasse T. Hansen**: Performed mammalian clonogenic assays and recombination assays. **Morten Grauslund: **Performed RNA purification and microarray analysis. **Peter B. Jensen**: Participated in planning and monitoring the study. **Maxwell Sehested: **Participated in the initiation and conduction of the study. All authors read and approved the final manuscript.

## Supplementary Material

Additional file 1 Clonogenic sensitivity of mutant single-gene deletion yeast strains towards ICRF-187 and m-AMSA. Clonogenic sensitivity of a panel of human topoisomerase II α-transformed haploid yeast deletion strains towards equitoxic (to wt cells) concentrations of ICRF-187 and m-AMSA. Error-bars represent SEM of 3 – 10 independent experiments. Click here for file

Additional file 2 Level of killing of yeast cells used in transcriptional profiling experiments. Fresh colonies of pMJ1-transformed JN362A_t2–4 _cells were inoculated into YPD medium and grown overnight at 34°C, 150 rpm. The cultures were then diluted into 50 ml YPD medium to obtain an OD_600 _of 0.2. After growing the cells for 2 hours to assure exponential growth, equitoxic concentrations of ICRF-187 and m-AMSA were applied and the cells were grown for an additional 2 hours before RNA was isolated. The figure depicts the clonogenecity of drug treated and untreated cells. Exposure of the cells to the two drugs resulted in a reduction of their clonogenecity of approx. 50 %. Error bars-represent SEM of three independent experiments. Click here for file

Additional file 3 Transcriptional response towards ICRF-187. A list of yeast genes whose average expression in two independent experiments is induced or repressed more than 1.5 fold by exposure to ICRF-187. Click here for file

Additional file 4 Transcriptional response towards m-AMSA. A list of yeast genes whose average expression in two independent experiments is induced or repressed more than 1.5 fold by exposure to m-AMSA. Click here for file

Additional file 5 Topoisomerase II activity levels in hamster cell lines. The levels of topoisomerase II catalytic activity in crude extracts from wt and recombination defective hamster cell lines. Error-bars represent SEM of two independent experiments. No difference in the level of topoisomerase II catalytic (DNA strand passage activity) is observed. Click here for file
