# Supplementary figures and images for: Characterisation of cytotoxicity and DNA damage induced by the topoisomerase II-directed bisdioxopiperazine anti-cancer agent ICRF-187 (dexrazoxane) in yeast and mammalian cells
Source: BMC Pharmacol. 2004 Dec 2;4:31. doi: 10.1186/1471-2210-4-31 (PMC545072; doi:10.1186/1471-2210-4-31)

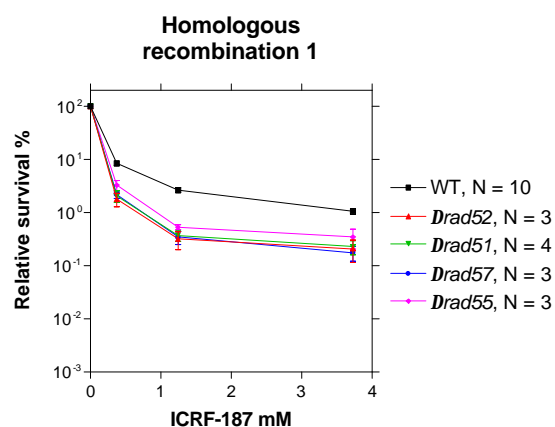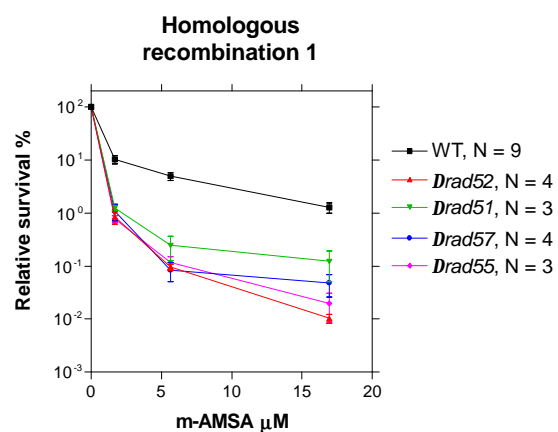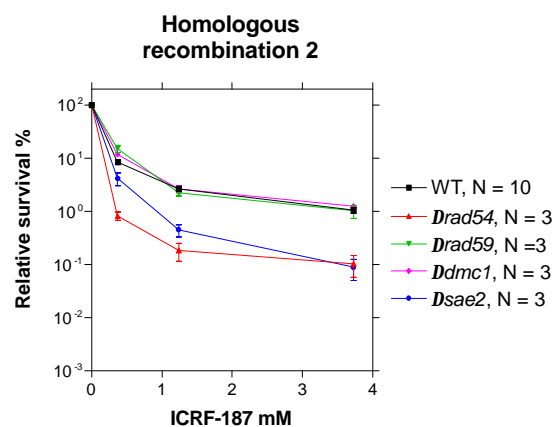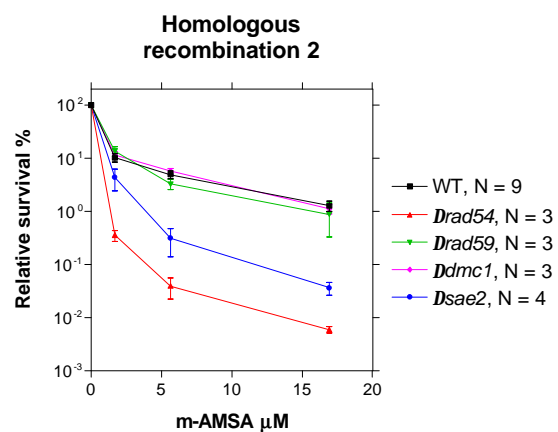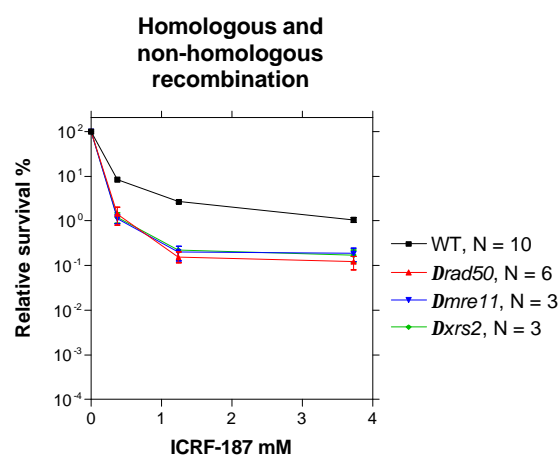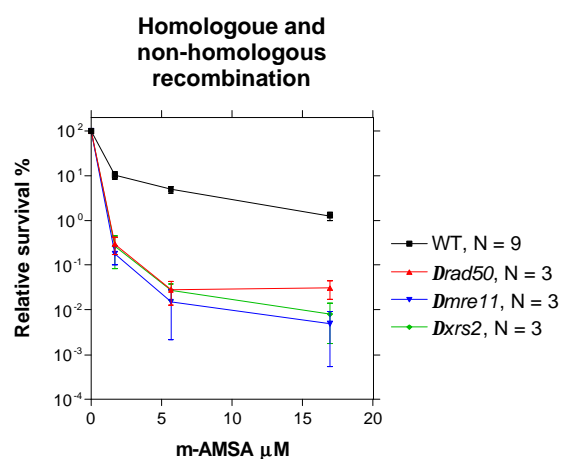

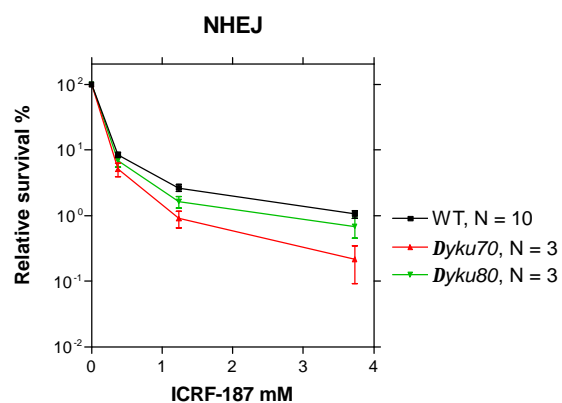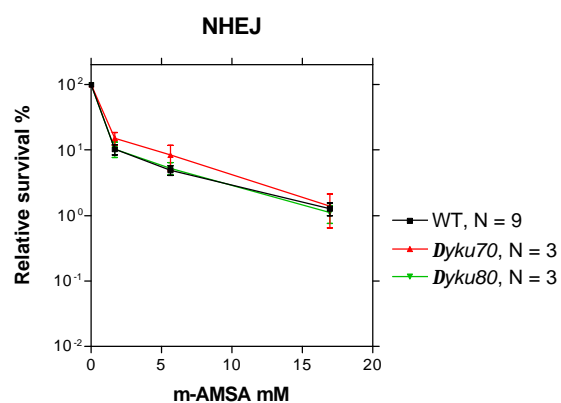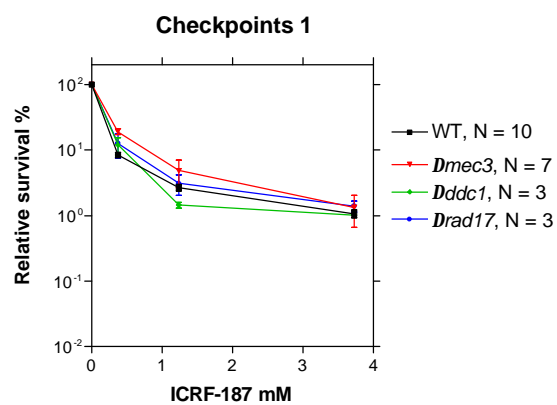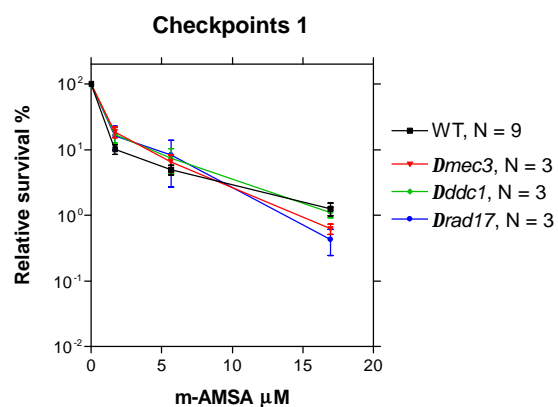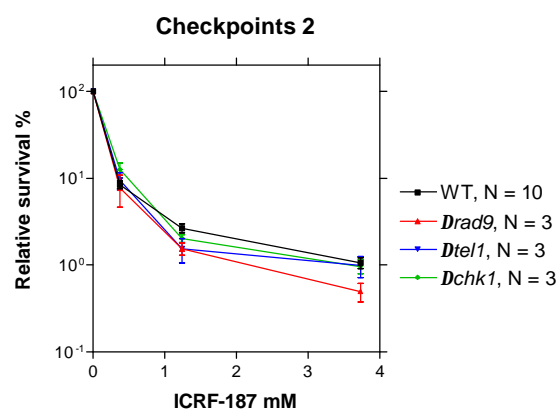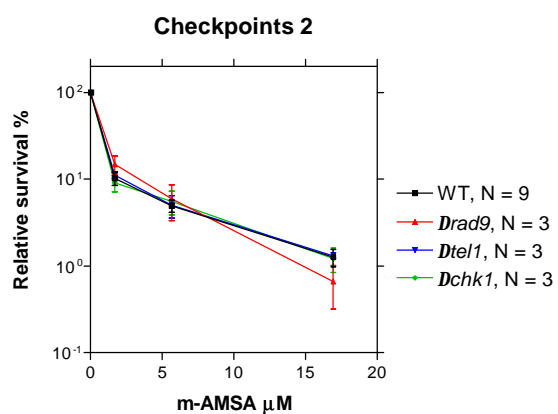

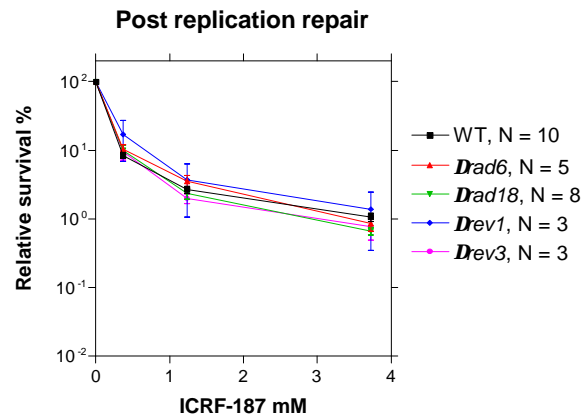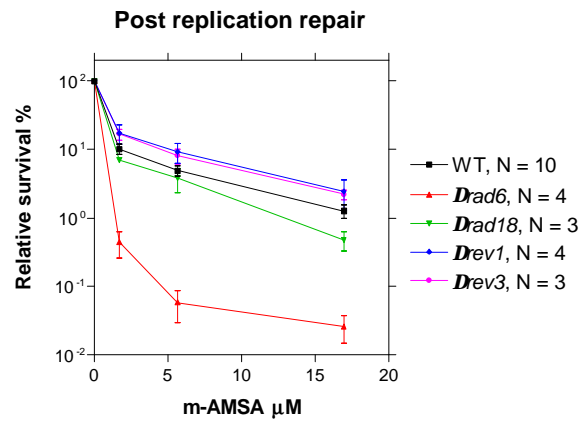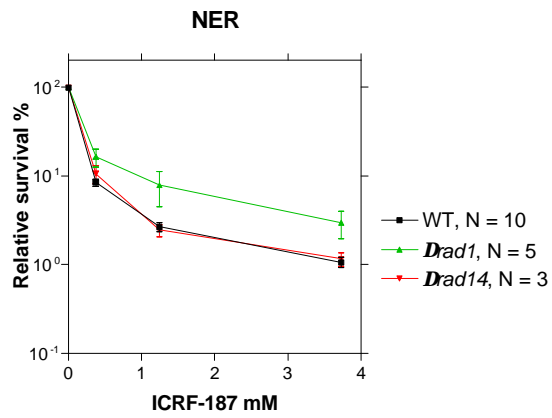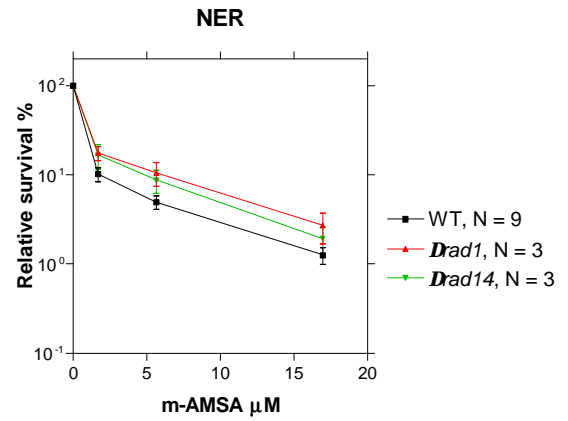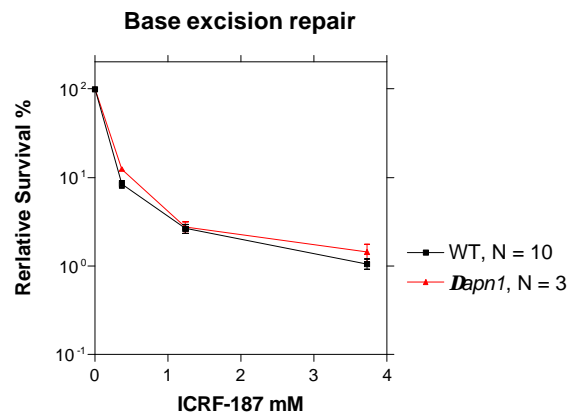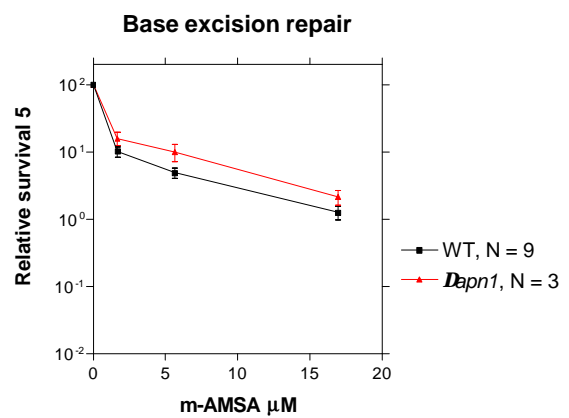

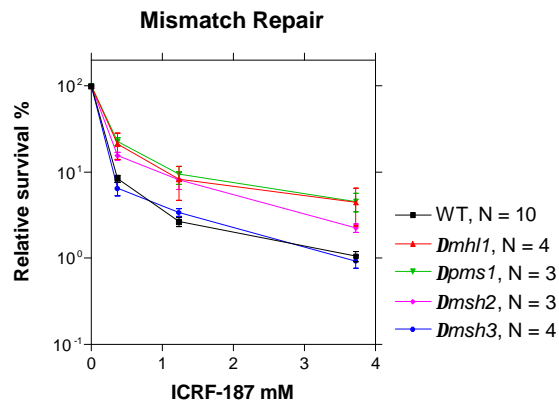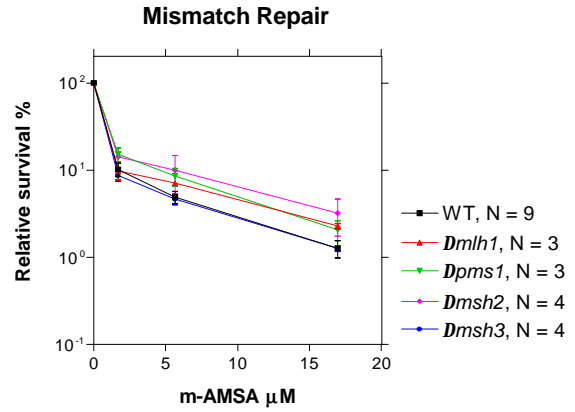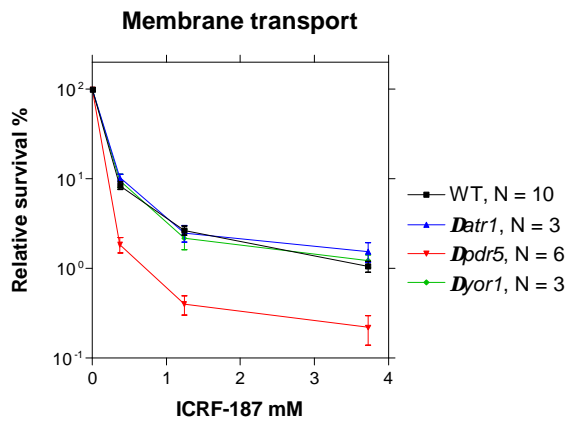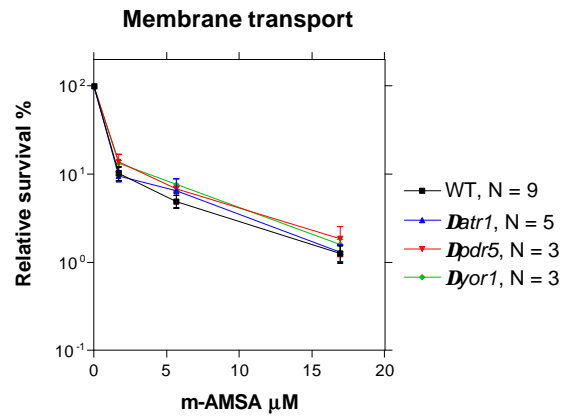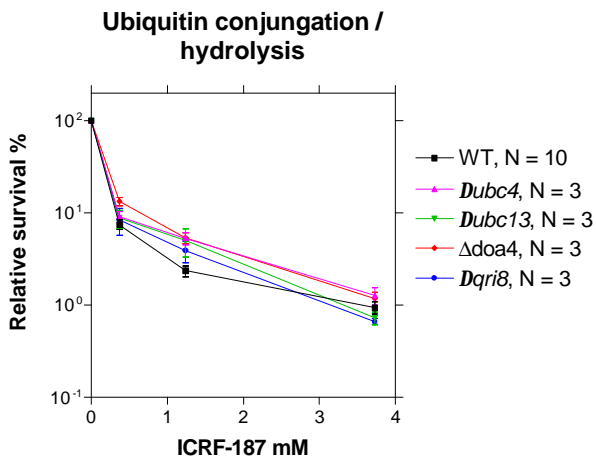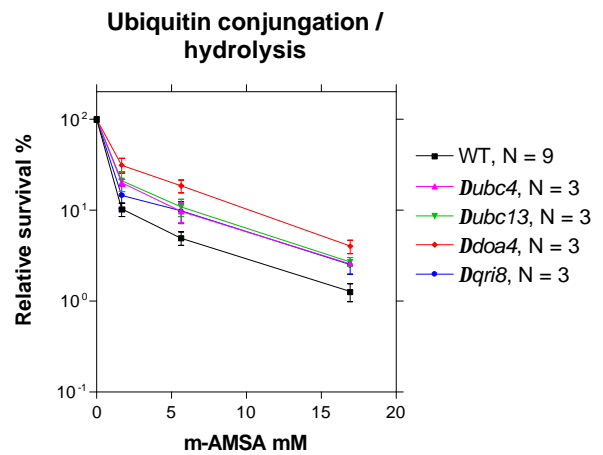

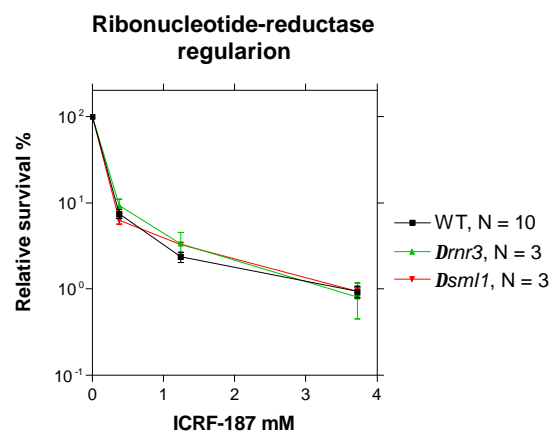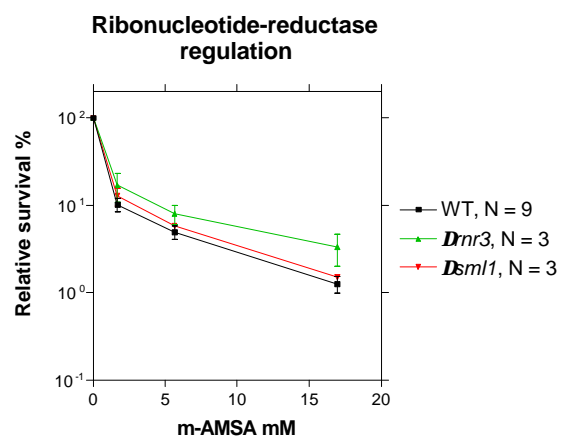

Supplement: Additional file 1 — Clonogenic sensitivity of mutant single-gene deletion yeast strains towards ICRF-187 and m-AMSA. Clonogenic sensitivity of a panel of human topoisomerase II α-transformed haploid yeast deletion strains towards equitoxic (to wt cells) concentrations of ICRF-187 and m-AMSA. Error-bars represent SEM of 3 – 10 independent experiments. [file 1471-2210-4-31-S1.pdf]

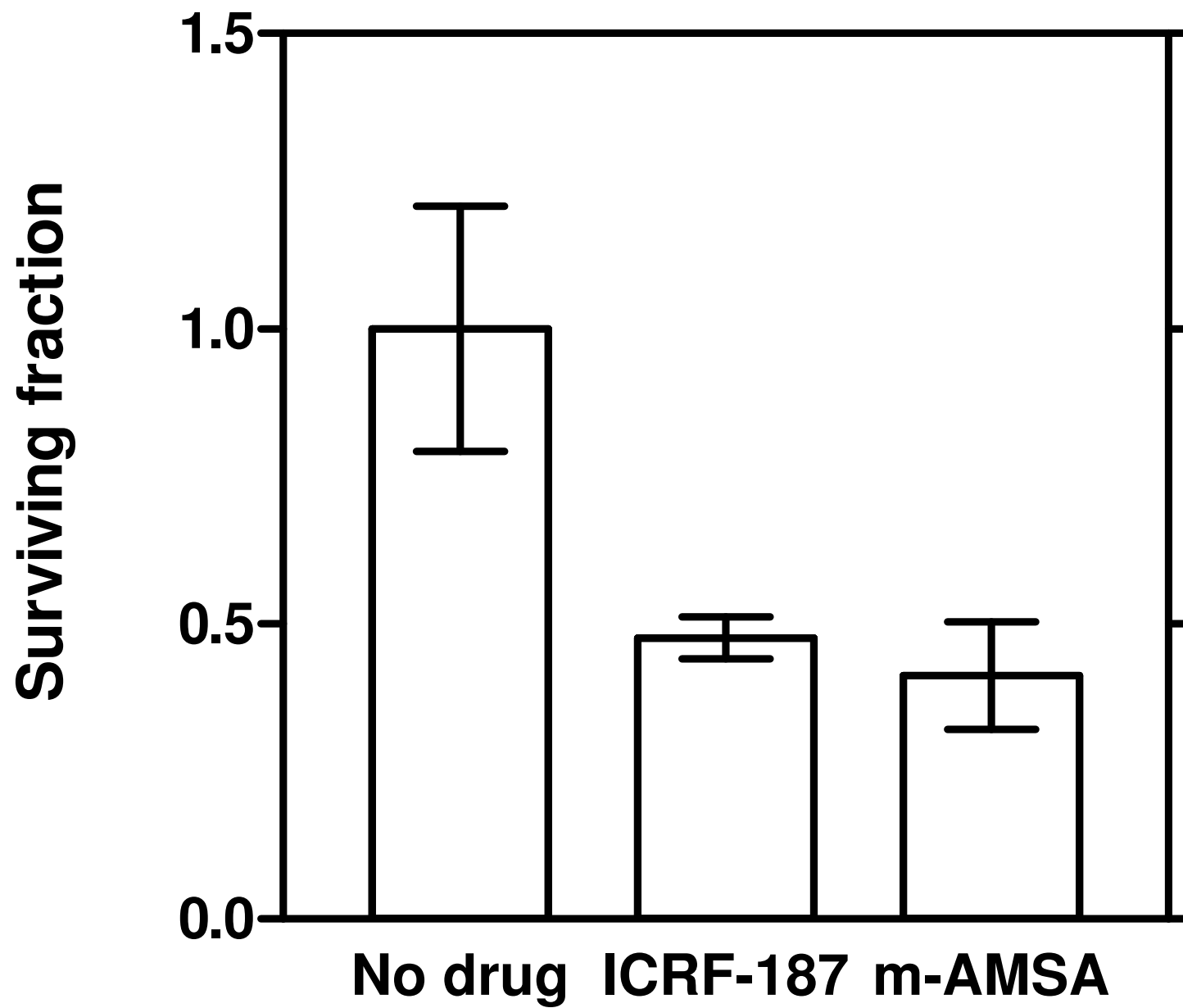

Supplement: Additional file 2 — Level of killing of yeast cells used in transcriptional profiling experiments. Fresh colonies of pMJ1-transformed JN362At2–4 cells were inoculated into YPD medium and grown overnight at 34°C, 150 rpm. The cultures were then diluted into 50 ml YPD medium to obtain an OD600 of 0.2. After growing the cells for 2 hours to assure exponential growth, equitoxic concentrations of ICRF-187 and m-AMSA were applied and the cells were grown for an additional 2 hours before RNA was isolated. The figure depicts the clonogenecity of drug treated and untreated cells. Exposure of the cells to the two drugs resulted in a reduction of their clonogenecity of approx. 50 %. Error bars-represent SEM of three independent experiments. [file 1471-2210-4-31-S2.pdf]

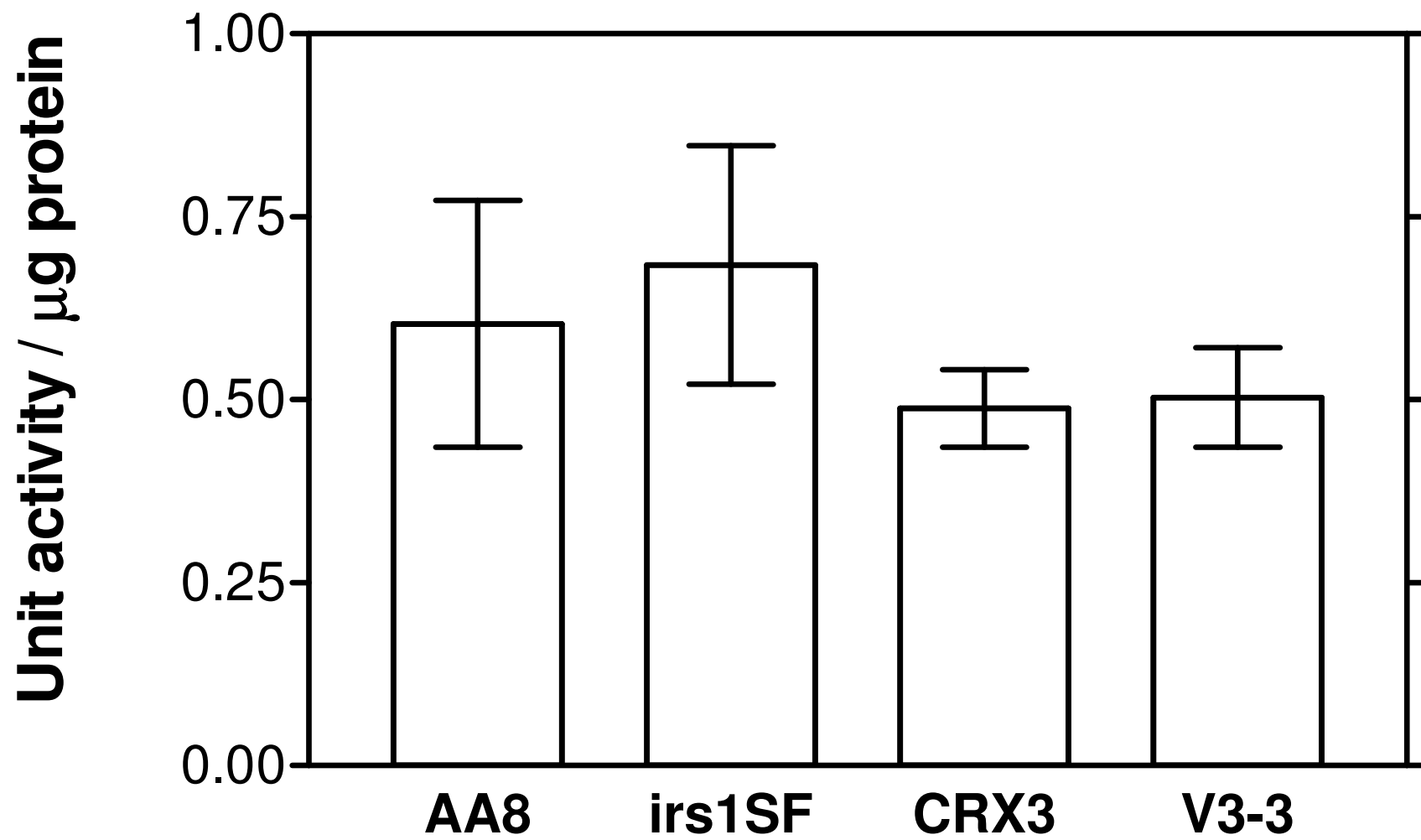

Supplement: Additional file 5 — Topoisomerase II activity levels in hamster cell lines. The levels of topoisomerase II catalytic activity in crude extracts from wt and recombination defective hamster cell lines. Error-bars represent SEM of two independent experiments. No difference in the level of topoisomerase II catalytic (DNA strand passage activity) is observed. [file 1471-2210-4-31-S5.pdf]
