# Supplementary material for: Characterisation of cytotoxicity and DNA damage induced by the topoisomerase II-directed bisdioxopiperazine anti-cancer agent ICRF-187 (dexrazoxane) in yeast and mammalian cells
Source: BMC Pharmacol. 2004 Dec 2;4:31. doi: 10.1186/1471-2210-4-31 (PMC545072; doi:10.1186/1471-2210-4-31)
Supplement: Additional file 3 — Transcriptional response towards ICRF-187. A list of yeast genes whose average expression in two independent experiments is induced or repressed more than 1.5 fold by exposure to ICRF-187. [file 1471-2210-4-31-S3.pdf]

Gene	ORF	Description	Control exp. 1	Control exp. 2	Control mean	ICRF187 exp. 2	ICRF187 exp. 1	ICRF187 mean	Fold change
RDN37-1	RDN37-1	RDN37-1 35S ribosomal RNA	837,74	337,52	566,17	3993,91	565,77	2278,04	4,02
Q0155	Q0155	Similarity to *Sauroleishmania* NADH dehydrogenase (ubiquinone) chain 5 Found forward in NC_001224 between 3940 and 4167 with 99.122807% identity.	74,64	22,77	48,72	282,23	35,29	158,78	3,26
YGR237C	YGR237C	YGR237C weak similarity to YOR019w	47,58	39,43	43,27	218,44	57,45	137,96	3,19
RNR3	YIL066C	Ribonucleotide reductase (ribonucleoside-diphosphate reductase) large subunit	158,36	139,58	148,41	655,05	290,5	472,91	3,19
HUG1	YML058W-A	Protein involved in the Mec1p-mediated checkpoint pathway that responds to DNA damage or replication arrest, transcription is induced by DNA damage	344,24	271,71	308,46	947,05	988,83	965,65	3,13
YKL071W	YKL071W	YKL071W weak similarity to A.parasiticus nor-1 protein	296,81	169,24	232,93	778,65	560,15	669,61	2,87
HSP12	YFL014W	12 kDa heat shock protein	217,95	289,95	254,13	537,43	866,77	702,09	2,76
SNR6	SNR6	SNR6 snRNA	135,92	144,45	140,01	384,22	369,82	377,56	2,7
YHR140W	YHR140W	YHR140W hypothetical protein	40,44	27,19	33,86	87,98	75,2	81,48	2,41
PDR12	YPL058C	Multidrug resistance transporter	340,85	326,07	332,93	804,67	770,45	788,22	2,37
SOL4	YGR248W	Similar to SOL3	36,92	49,86	43,72	64,53	137,88	101,18	2,31
TM(CAU)J1	TM(CAU)J1	TM(CAU)J1 tRNA-Met	17,21	11,21	14,36	37,89	27,3	32,64	2,27
YLL056C	YLL056C	YLL056C weak similarity to Y.pseudotuberculosis CDP-3,6-dideoxy-D-glycero-L-glycero-4-hexulose-5-epimerase	141,03	168,61	155,32	327,99	365,73	347,08	2,23
OYE3	YPL171C	NAD(P)H dehydrogenase	108,09	116,58	111,89	215,39	283,88	249,64	2,23
TK(CUU)J	TK(CUU)J	TK(CUU)J tRNA-Lys	55,12	50,26	52,75	135,7	99,76	117,76	2,23
TR(ACG)L	TR(ACG)L	TR(ACG)L tRNA-Arg	83,2	172,74	127,95	210,56	325,88	268,21	2,1
TA(UGC)A	TA(UGC)A	TA(UGC)A tRNA-Ala	205,59	199,18	202,04	487,55	353,2	420,33	2,08
GAD1	YMR250W	Glutamate decarboxylase	78,4	155,1	116,79	172,01	309,77	240,76	2,06
HXK1	YFR053C	Hexokinase I (PI) (also called Hexokinase A)	183,81	444,26	314,09	438,13	856,27	647,33	2,06
TR(ACG)O	TR(ACG)O	TR(ACG)O tRNA-Arg	70,38	179,39	124,95	179,08	332,86	255,96	2,05
YRO2	YBR054W	Homolog to HSP30 heat shock protein YRO1 (S. cerevisiae) 7	313,99	551,54	433,65	663,69	1090,62	876,97	2,02
TR(ACG)K	TR(ACG)K	TR(ACG)K tRNA-Arg	96,28	177,44	136,89	214,64	327,35	271,07	1,98
TA(UGC)O	TA(UGC)O	TA(UGC)O tRNA-Ala	135,84	110,2	122,76	279,51	201,2	240,21	1,96

NNL072W	NNL072W	Non-annotated SAGE orf Found forward in NC_001146 between 89212 and 89394 with 100% identity. See citation Velculescu, V.E., et al. (1997) Characterization of the yeast transcriptome. Cell 8:243-251	34,22	34,34	34,28	60	74,37	67,02	1,95
PDR15	YDR406W	Probable multidrug resistance transporter	171,42	174,79	172,47	344,7	329,69	337,03	1,95
YOL053C	YOL053C	YOL053C DNA Damage Responsive	559,75	1066,58	813,71	981,05	2166,9	1574,19	1,93
TS(UGA)E	TS(UGA)E	TS(UGA)E tRNA-Ser	403,33	368,61	385,11	813,27	671,12	741,72	1,93
TR(ACG)J	TR(ACG)J	TR(ACG)J tRNA-Arg	96,47	189,83	143,11	177,05	371,7	274,4	1,92
GAL5	YMR105C	Phosphoglucomutase	125,13	160,31	142,59	194,45	340,13	267,24	1,87
YCL042W	YCL042W	YCL042W questionable ORF	335,83	662,65	499,05	810,91	1051	931,29	1,87
RAD51	YER095W	RecA homolog\; Rad51p colocalizes to ~ 65 spots with Dmc1p prior to synapsis (independently of ZIP1 and DMC1), and interacts with Rad52p and Rad55p\; human Rad51p homolog interacts with Brca2 protein which has been implicated in causing breast cancer	698,86	712,21	704,54	1305,8	1319,92	1312,6	1,86
PNC1	YGL037C	Pyrazinamidase and nicotinamidase	535,05	959,55	747,64	1149,31	1635,85	1392,06	1,86
gGR14	gGR14	*Saccharomyces cerevisiae* chromosome VII, complete chromosome sequence. Found forward in NC_001139 between 1054406 and 1055405 with 100% identity.	229,75	301,45	265,58	542,03	433,77	487,88	1,84
HXT9	YJL219W	Hexose permease	26,34	31,53	28,82	43,85	61,35	52,52	1,82
NDR010C	NDR010C	Non-annotated SAGE orf Found reverse in NC_001136 between 541225 and 541422 with 100% identity.	92,81	74,47	83,79	153,25	152,1	152,76	1,82
TS(UGA)P	TS(UGA)P	TS(UGA)P tRNA-Ser	459,16	447,39	452,79	862,96	789,49	825,93	1,82
RDN37-1	RDN37-1	RDN37-1 35S ribosomal RNA	435,73	659,71	548,22	1102,44	883,31	992,66	1,81
YPL014W	YPL014W	YPL014W hypothetical protein	245,91	213,59	230,61	442,42	381,84	412,52	1,79
STF2	YGR008C	ATPase stabilizing factor	272,5	308,53	290,38	475,51	561,33	518,56	1,79
SPO4	YOR273C	YOR273C similarity to resistance proteins	145,01	152,78	148,97	284,1	244,29	264,65	1,78
YJL163C	YJL163C	YJL163C hypothetical protein	100,86	130,04	114,82	224,48	181,5	202,66	1,76
TFS1	YLR178C	(putative) lipid binding protein\; supressor of a cdc25 mutation	263,12	416,43	339,84	425,59	769,94	597,71	1,76
YGR035C	YGR035C	YGR035C hypothetical protein	233,3	134,44	184,06	412,03	235,4	323,72	1,76
YJL103C	YJL103C	YJL103C putative regulatory protein	10,75	17,11	14,16	20,07	28,99	24,64	1,74
GRE2	YOL151W	putative reductase	841,44	818,45	834,09	1524,29	1381,35	1453	1,74

YCR062W	YCR062W	YCR062W similarity to Ytp1p protein	237,37	257,06	247,05	438,24	424,17	430,83	1,74
gGR14	gGR14	*Saccharomyces cerevisiae* chromosome VII, complete chromosome sequence. Found forward in NC_001139 between 1053406 and 1054405 with 100% identity.	113,2	127,36	120,41	208,93	209,55	209,3	1,74
GPH1	YPR160W	Glycogen phosphorylase	65,66	83,56	74,41	120,39	137,3	128,82	1,73
PRX1	YBL064C	Similar to thiol-specific antioxidant enzymes such as rehydrin\/peroxiredoxin	171,99	274,82	223,3	316,53	455,67	386,16	1,73
HSP26	YBR072W	Heat shock protein 26	664,86	1215,8	939,31	1184,01	2064,85	1624,33	1,73
TR(UCU)K	TR(UCU)K	TR(UCU)K tRNA-Arg	76,47	65,16	70,77	129,79	114,9	122,5	1,73
TPO1	YLL028W	Polyamine transport protein	1144,28	1095,06	1123,29	1999,22	1859,25	1927,38	1,72
MSC1	YML128C	YML128C C-terminal part starting with aa 262 cause growth inhibition when overexpressed	106,4	134,13	120,32	153,35	259,45	206,38	1,72
SHC1	YER096W	Sporulation-specific homolog of csd4	29,28	31,93	30,66	57,93	47,48	52,84	1,72
NER027C	NER027C	Non-annotated SAGE orf Found reverse in NC_001137 between 311731 and 311907 with 100% identity.	22,2	13,5	17,75	32,4	28,48	30,54	1,72
RDN37-1	RDN37-1	RDN37-1 35S ribosomal RNA	372,09	398,65	384,29	877,39	443,3	660,3	1,72
gOR05	gOR05	*Saccharomyces cerevisiae* chromosome XV, complete chromosome sequence. Found forward in NC_001147 between 620016 and 621015 with 100% identity.	346,39	384,56	363,62	662,03	592,33	626,72	1,72
YCR061W	YCR061W	YCR061W hypothetical protein	411,03	498,46	454,38	779,34	772,34	775,62	1,71
SNR13	SNR13	SNR13 snRNA	188,75	401,84	295,17	441,02	561,23	501,41	1,7
ECM29	YHL030W	Involved in cell wall biogenesis	59,22	81,94	70,61	107,22	132,94	120,08	1,7
SKF1	YKL051W	YKL051W hypothetical protein	316,39	327,51	322,28	493,12	597,73	545,61	1,69
RNY1	YPL123C	Ribonuclease from the T2 family of ribonucleases	51,02	60,03	55,49	88,03	99,63	93,79	1,69
TR(UCU)D	TR(UCU)D	TR(UCU)D tRNA-Arg	74,9	67,45	70,96	119,91	119,33	119,6	1,69
GUP2	YPL189W	Putative active glycerol transporter	29,86	31,55	30,56	52,57	50,1	51,43	1,68
RPP1B	YDL130W	Ribosomal protein P1B (L44') (YP1beta) (Ax)	280,58	380,39	329,8	485,91	622,38	553,95	1,68
YOL150C	YOL150C	YOL150C questionable ORF	11,49	14,64	13,19	20,74	23,32	21,97	1,67
YCR061W	YCR061W	YCR061W hypothetical protein	509,34	703,29	606,4	999,05	1025,5	1011,11	1,67
YHR138C	YHR138C	YHR138C hypothetical protein	415,71	423,1	419,31	681,65	722,32	700,87	1,67
NKL038W	NKL038W	Non-annotated SAGE orf Found forward in NC_001143 between 94073 and 94228 with 100% identity.	39,1	25,73	32,19	48,55	58,12	53,42	1,66

GPP2	YER062C	DL-glycerol-3-phosphatase	237,46	389,36	313,18	471,33	570,34	520,61	1,66
YKL070W	YKL070W	YKL070W similarity to B.subtilis transcriptional regulatory protein	30,58	24,01	27,24	52,53	37,49	44,96	1,65
GPM2	YDL021W	Phosphoglycerate mutase, involved in glycolysis	65,92	83,86	74,86	118,41	128,72	123,56	1,65
AFR1	YDR085C	Cytoskeletal protein, similar to arrestins	30,61	42,12	36,23	57,41	62,42	59,72	1,65
RPR1	RPR1	RPR1 RNase P RNA	194,99	271	233,15	392,81	376,28	385,33	1,65
RAD54	YGL163C	DNA-dependent ATPase	70,64	67,61	69,39	119,9	108,17	114,27	1,65
NOR009W	NOR009W	Non-annotated SAGE orf Found forward in NC_001147 between 464469 and 464630 with 100% identity.	209,39	258,5	234,32	382,67	384,74	383,8	1,64
YBR026C	YBR026C	Nuclear protein that binds to T-rich strand of core consensus sequence of autonomously replicating sequence	87,2	92,09	89,34	134,58	158,51	146,66	1,64
TT(UGU)P	TT(UGU)P	TT(UGU)P tRNA-Thr	39,94	44,49	41,52	67,76	68,3	68,01	1,64
gOR05	gOR05	*Saccharomyces cerevisiae* chromosome XV, complete chromosome sequence. Found forward in NC_001147 between 619016 and 620015 with 100% identity.	54,23	54,49	54,36	103,29	74,64	89,1	1,64
YMR090W	YMR090W	YMR090W strong similarity to B. subtilis conserved hypothetical protein yhfK	251	306,02	278,66	360,44	550,94	455,3	1,63
YPC1	YBR183W	Alkaline ceramidase with reverse activity	248,8	325,77	287,15	400,08	538,14	469,13	1,63
NDR082C	NDR082C	Non-annotated SAGE orf Found reverse in NC_001136 between 971619 and 971783 with 100% identity.	53,5	50,1	51,95	72,41	97,46	84,81	1,63
XBP1	YIL101C	Transcriptional repressor	77	104,32	90,81	133,74	162,06	147,96	1,63
TQ(UUG)D1	TQ(UUG)D1	TQ(UUG)D1 tRNA-Gln	76,99	62,71	69,77	102,93	125,01	113,44	1,63
gJL01	gJL01	*Saccharomyces cerevisiae* chromosome X, complete chromosome sequence. Found forward in NC_001142 between 13138 and 14137 with 100% identity.	5,74	5,34	5,49	10,44	7,47	8,95	1,63
SNR37	SNR37	SNR37 small nuclear RNA37	49,48	126,27	87,96	101,04	183,53	142,37	1,62
RSB1	YOR049C	YOR049C similarity to YER185w, Rta1p	371,97	263,59	319,32	562,46	470,04	512,81	1,61
YOR161C	YOR161C	YOR161C similarity to C.elegans cosmid F35C8	138,12	146,27	142,57	201	258,96	229,9	1,61
DCS2	YOR173W	YOR173W strong similarity to YLR270w	31,41	69,27	50,5	57,57	105,18	81,36	1,61
RDN37-1	RDN37-1	RDN37-1 35S ribosomal RNA	137,91	131,7	135,05	392,2	41,95	217,06	1,61
TQ(UUG)E2	TQ(UUG)E2	TQ(UUG)E2 tRNA-Gln	86,03	71,94	79,24	115,99	139,94	127,83	1,61

GCY1	YOR120W	Similar to mammalian aldo\/keto reductases	128,04	206,77	167,25	184,06	352,79	268,35	1,6
FMP16	YDR070C	YDR070C hypothetical protein	59,85	84,11	71,76	86,86	142,11	114,62	1,6
YGL157W	YGL157W	YGL157W similarity to V.vinifera dihydroflavonol 4-reductase	273,25	359,23	316,11	481,81	532,09	507,25	1,6
NGR125W	NGR125W	Non-annotated SAGE orf Found forward in NC_001139 between 1057363 and 1057593 with 100% identity.	383,82	324,11	354,29	635,1	499,62	567,43	1,6
YHL044W	YHL044W	YHL044W similarity to subtelomeric encoded proteins	62,76	62,61	62,7	92,87	108,34	100,39	1,6
YHR087W	YHR087W	YHR087W hypothetical protein	260,89	342,59	301,29	406,31	560,2	482,93	1,6
YCR102C	YCR102C	YCR102C Alcohol dehydrogenase	118,89	136,23	127,46	202,64	202,22	202,44	1,59
STP4	YDL048C	Involved in tRNA splicing	166,41	138,86	152,84	230,76	256,37	243,42	1,59
VPS73	YGL104C	YGL104C similarity to glucose transport proteins	62,02	83,24	72,72	103,16	127,91	115,55	1,59
SDP1	YIL113W	YIL113W strong similarity to dual-specificity phosphatase Msg5p	92,66	135,7	114,23	195,08	167,78	181,93	1,59
SWI1	YPL016W	Zinc-finger transcription factor	73,47	76,73	75,52	130,39	107,15	119,09	1,58
TR(UCU)E	TR(UCU)E	TR(UCU)E tRNA-Arg	63,19	52,87	57,94	98,25	84,26	91,29	1,58
YDR533C	YDR533C	YDR533C strong similarity to hypothetical proteins YPL280w, YOR391c and YMR322c	1808,8	2546,62	2176,29	3231,87	3568,01	3402,46	1,56
WSC4	YHL028W	Putative integral membrane protein containing novel cysteine motif. Similarity to SLG1 (WSC1), WSC2 and WSC3	92,8	96,84	94,74	137,92	156,32	147,37	1,56
NIR010W	NIR010W	Non-annotated SAGE orf Found forward in NC_001141 between 425520 and 425678 with 100% identity.	11,7	13,27	12,54	20,69	18,38	19,53	1,56
TR(UCU)J1	TR(UCU)J1	TR(UCU)J1 tRNA-Arg	77,78	59,27	68,69	110,06	104,86	107,49	1,56
gGR14	gGR14	*Saccharomyces cerevisiae* chromosome VII, complete chromosome sequence. Found forward in NC_001139 between 1055406 and 1056405 with 100% identity.	289,6	371,11	328,26	515,77	512,01	513,68	1,56
SNR3	SNR3	SNR3 small nuclear RNA3	32,03	69,7	50,88	59,79	98,23	78,98	1,55
YNL134C	YNL134C	YNL134C similarity to C.carbonum toxD gene	1632,12	2324,71	1978,39	2821,6	3332,65	3074,47	1,55
FUN34	YNR002C	Putative transmembrane protein	104,14	118,15	111,04	162,08	180,84	171,6	1,55
YCL049C	YCL049C	YCL049C hypothetical protein	308,74	335,24	321,58	500,63	499,1	499,77	1,55
INH1	YDL181W	ATPase inhibitor	193,2	229,76	211,31	277,28	376,36	326,77	1,55
GPD1	YDL022W	Glycerol-3-phosphate dehydrogenase	782,25	1145,29	963,3	1367,76	1622,29	1495,94	1,55

HSP42	YDR171W	Heat shock protein similar to HSP26, involved in cytoskeleton assembly	243,6	341,77	292,43	349,42	549,67	449,39	1,54
YJL132W	YJL132W	YJL132W weak similarity to human phospholipase D	70,79	68,67	69,82	110,94	102,61	106,62	1,53
RNR2	YJL026W	Small subunit of ribonucleotide reductase	1759,16	2025,83	1890,2	2842,11	2954,57	2898,45	1,53
UBP1	YKR098C	Ubiquitin-specific protease	52,95	48,32	50,51	87,71	66,33	77,07	1,53
SNR38	SNR38	SNR38 snRNA	181,38	143,36	162,42	309,69	187,3	248,49	1,53
SNR57	SNR57	SNR57 snRNA	33,3	27,44	30,48	40,65	52,66	46,52	1,53
NBR045C	NBR045C	Non-annotated SAGE orf Found reverse in NC_001134 between 624453 and 624656 with 100% identity.	433,1	322,1	377,46	629,94	523,16	576,84	1,53
YDL085C	YDL085C	YDL085C identified by SAGE	481,11	482,5	481,54	731,7	745,47	738,33	1,53
TH(GUG)K	TH(GUG)K	TH(GUG)K tRNA-His	117,71	106,35	111,65	174,69	167,03	170,73	1,53
TH(GUG)M	TH(GUG)M	TH(GUG)M tRNA-His	112,05	106,17	109,07	155,86	177,13	166,55	1,53
SNR52	SNR52	SNR52 snRNA	161,73	117,84	139,97	229,67	198,8	213,15	1,52
TS(AGA)M	TS(AGA)M	TS(AGA)M tRNA-Ser	151,88	149,28	150,87	228,11	231,21	229,36	1,52
TC(GCA)P1	TC(GCA)P1	TC(GCA)P1 tRNA-Cys	107,14	61,05	84,47	139,65	117,47	128,37	1,52
YJL161W	YJL161W	YJL161W hypothetical protein	124,18	197,31	160,76	183,53	301,9	242,73	1,51
TR(UCU)M2	TR(UCU)M2	TR(UCU)M2 tRNA-Arg	72,43	53,46	62,87	111,91	77,18	94,73	1,51
TR(UCU)M1	TR(UCU)M1	TR(UCU)M1 tRNA-Arg	93,42	63,82	78,58	135,26	102,49	118,93	1,51
TE(CUC)D	TE(CUC)D	TE(CUC)D tRNA-Glu	382,4	328,22	355,21	617,31	458,47	537,79	1,51
YMR196W	YMR196W	YMR196W hypothetical protein	76,06	133,12	104,57	117,51	197,06	157,25	1,5
YGP1	YNL160W	YGP1 encodes gp37, a glycoprotein synthesized in response to nutrient limitation which is homologous to the sporulation-specific SPS100 gene	2302,44	3019,07	2661,21	3763,83	4220,91	3992,22	1,5
YNL115C	YNL115C	YNL115C weak similarity to S.pombe hypothetical protein SPAC23C11	107,84	124,56	116,18	151	197,84	174,34	1,5
YOL048C	YOL048C	YOL048C similarity to YAL018c and YOL047c	147,31	231,98	189,76	273,96	296,97	285,33	1,5
PHM8	YER037W	YER037W strong similarity to hypothetical protein YGL224c	103,32	163,29	133,13	170,5	228,81	199,79	1,5
TS(AGA)D2	TS(AGA)D2	TS(AGA)D2 tRNA-Ser	146,51	153,02	149,87	228,7	222,25	225,28	1,5
gGR14	gGR14	*Saccharomyces cerevisiae* chromosome VII, complete chromosome sequence. Found forward in NC_001139 between 1056406 and 1056753 with 100% identity.	1115,92	1352,88	1232,03	1933,44	1782,09	1850,61	1,5
YDL179W	YDL179W	Cyclin	177,36	208,62	192,74	112,87	144,27	128,27	-1,5
YLR042C	YLR042C	YLR042C hypothetical protein	109,22	128,37	118,94	66,83	90,3	78,55	-1,51

YOR264W	YOR264W	YOR264W hypothetical protein	147,23	173,41	160,17	105,7	106,43	106,19	-1,51
YFR034C	YFR034C	myc-type helix-loop-helix transcription factor	59,09	65,98	62,05	45	35,96	41,12	-1,51
gAR04	gAR04	*Saccharomyces cerevisiae* chromosome I, complete chromosome sequence. Found forward in NC_001133 between 212649 and 213648 with 100% identity.	19,86	25,53	23,18	22,16	8,24	15,2	-1,52
NNL025C	NNL025C	Non-annotated SAGE orf Found reverse in NC_001146 between 519600 and 519773 with 100% identity.	24,04	18,34	21,37	12,32	15,44	14,01	-1,53
YLR136C	YLR136C	Homolog of mammalian TIS11	545,25	665,06	605,35	359,26	425,25	392,07	-1,54
YLR086W	YLR086W	SMC chromosomal ATPase family member	19,29	20,95	20	15,44	10,07	12,93	-1,55
YLR286C	YLR286C	Endochitinase	4473,91	3961,99	4228,04	2838,28	2590,93	2711,39	-1,56
YER145C	YER145C	Iron permease	1321,09	1266,88	1293,09	821,69	834,24	827,5	-1,56
YGR012W	YGR012W	YGR012W similarity to E.nidulans cysteine synthase	139,79	149,77	144,51	120,06	64,6	92,32	-1,57
YLR214W	YLR214W	Ferric (and cupric) reductase	707,76	918,27	812,84	421,98	609,93	515,89	-1,58
BOP2	YLR267W	Bypass of PAM1	167,81	243,63	205,67	114,73	144,81	129,77	-1,58
NLR118C	NLR118C	Non-annotated SAGE orf Found reverse in NC_001144 between 708168 and 708338 with 100% identity.	87,76	61,34	74,92	44,03	50,03	47,28	-1,58
PDH1	YPR002W	prpD homolog (ie the propionate operon of many prokaryotes and specifically, the gene called prpD in that operon) This gene may be regarded as *real* in yeast by two criteria: it has close homologs (some known to be essential for propionate utilization) i	102,38	106,14	103,84	63,06	68,57	65,41	-1,59
ARN2	YHL047C	Triacetylfusarinine C transporter	902,13	907,44	904,53	691,72	425,61	558,83	-1,62
SNZ1	YMR096W	Encodes highly conserved 35 kDa protein that shows increased expression after entry into stationary phase	1177,46	1737,05	1457,15	800,22	961,31	881,26	-1,65
YOR315W	YOR315W	YOR315W hypothetical protein	345,11	359,73	352,09	248,39	170,82	209,65	-1,68
gKR06	gKR06	*Saccharomyces cerevisiae* chromosome XI, complete chromosome sequence. Found forward in NC_001143 between 624375 and 624566 with 100% identity.	9,59	9,39	9,5	7,33	3,69	5,67	-1,68
DUR3	YHL016C	Urea transporter	201,42	234,87	218,12	114,47	142,13	128,23	-1,7
HXT2	YMR011W	High affinity hexose transporter-2	2183,56	2359,09	2270,86	1304,95	1355,31	1330,82	-1,71

NGR003C	NGR003C	Non-annotated SAGE orf Found reverse in NC_001139 between 536199 and 536372 with 100% identity.	18,62	19,46	18,9	9,87	12,24	10,98	-1,72
GSY2	YLR258W	Glycogen synthase (UDP-gluocse--starch glucosyltransferase)	354,67	474,42	414,6	217,9	254,53	236,21	-1,76
FRE2	YOR384W	Similar to FRE2	25,93	56,29	41,06	20,81	25,27	23,17	-1,77
gAR04	gAR04	*Saccharomyces cerevisiae* chromosome I, complete chromosome sequence. Found forward in NC_001133 between 210649 and 211648 with 100% identity.	29,75	19,08	24,27	19,35	7,12	13,73	-1,77
gAR03	gAR03	*Saccharomyces cerevisiae* chromosome I, complete chromosome sequence. Found forward in NC_001133 between 164789 and 165788 with 100% identity.	23,07	26,03	24,42	8,81	15,07	11,85	-2,06
