# Supplementary material for: Characterisation of cytotoxicity and DNA damage induced by the topoisomerase II-directed bisdioxopiperazine anti-cancer agent ICRF-187 (dexrazoxane) in yeast and mammalian cells
Source: BMC Pharmacol. 2004 Dec 2;4:31. doi: 10.1186/1471-2210-4-31 (PMC545072; doi:10.1186/1471-2210-4-31)
Supplement: Additional file 4 — Transcriptional response towards m-AMSA. A list of yeast genes whose average expression in two independent experiments is induced or repressed more than 1.5 fold by exposure to m-AMSA. [file 1471-2210-4-31-S4.pdf]

Gene	ORF	Description	Control exp.1	Control exp. 2	Control mean	mAMSA exp. 1	mAMSA exp. 2	mAMSA mean	Fold change
HUG1	YML058W-A	Suppressor of mec lethality	344,24	271,71	308,46	1693,75	1409,83	1551,51	**5,03**
RNR3	YIL066C	Ribonucleotide reductase (ribonucleoside-diphosphate reductase) large subunit	158,36	139,58	148,41	707,98	469,54	588,76	**3,97**
YOL053C	YOL053C	YOL053C DNA Damage Responsive	559,75	1066,58	813,71	1074,88	2448,41	1761,74	**2,17**
GAD1	YMR250W	Glutamate decarboxylase	78,4	155,1	116,79	194,86	308,25	251,43	**2,15**
AAD6	YFL056C	Hypothetical aryl-alcohol dehydrogenase (AAD)	80,51	73,96	77,25	186,93	143,25	165,09	**2,14**
HXK1	YFR053C	Hexokinase I (PI) (also called Hexokinase A)	183,81	444,26	314,09	392	943,7	667,91	**2,13**
YCL042W	YCL042W	YCL042W questionable ORF	335,83	662,65	499,05	788,55	1306,1	1047,02	**2,1**
OYE3	YPL171C	NAD(P)H dehydrogenase	108,09	116,58	111,89	213,37	255,06	233,78	**2,09**
YJL103C	YJL103C	YJL103C putative regulatory protein	10,75	17,11	14,16	29,52	27,19	28,32	**2**
NKL038W	NKL038W	Non-annotated SAGE orf Found forward in NC_001143 between 94073 and 94228 with 100% identity. See citation Velculescu, V.E., et al. (1997) Characterization of the yeast transcriptome. Cell 8:243-251	39,1	25,73	32,19	67,08	61,57	64,1	**1,99**
RAD51	YER095W	RecA homolog\; Rad51p colocalizes to ~ 65 spots with Dmc1p prior to synapsis (independently of ZIP1 and DMC1), and interacts with Rad52p and Rad55p\; human Rad51p homolog interacts with Brca2 protein which has been implicated in causing breast cancer	698,86	712,21	704,54	1312,55	1441,91	1376,77	**1,95**
RDN37-1	RDN37-1	RDN37-1 35S ribosomal RNA	837,74	337,52	566,17	1198,76	1002,92	1094,11	**1,93**
TFS1	YLR178C	(putative) lipid binding protein\; supressor of a cdc25 mutation	263,12	416,43	339,84	444,47	846,88	645,56	**1,9**
NBR045C	NBR045C	Non-annotated SAGE orf Found reverse in NC_001134 between 624453 and 624656 with 100% identity.	433,1	322,1	377,46	853,57	561,81	707,72	**1,87**
GAL5	YMR105C	Phosphoglucomutase	125,13	160,31	142,59	227,12	302,51	264,86	**1,86**
LAP4	YKL103C	Vacuolar aminopeptidase ysc1	265,13	386,72	325,96	497,43	677,37	587,38	**1,8**
HSP26	YBR072W	Heat shock protein 26	664,86	1215,8	939,31	1318,58	2065,19	1691,36	**1,8**
STP4	YDL048C	Involved in tRNA splicing	166,41	138,86	152,84	241,17	305,04	273,19	**1,79**
YHR140W	YHR140W	YHR140W hypothetical protein	40,44	27,19	33,86	66,41	54,15	60,2	**1,78**
RNR2	YJL026W	Small subunit of ribonucleotide reductase	1759,16	2025,83	1890,2	3574,73	3104,66	3339,67	**1,77**
GPM2	YDL021W	Phosphoglycerate mutase, involved in glycolysis	65,92	83,86	74,86	114,24	150,08	132,16	**1,77**
YHR138C	YHR138C	YHR138C hypothetical protein	415,71	423,1	419,31	683,91	793,8	739,2	**1,76**

YJL163C	YJL163C	YJL163C hypothetical protein	100,86	130,04	114,82	209,62	192,03	200,81	**1,75**
ARG3	YJL088W	Ornithine carbamoyltransferase	56,76	66,72	61,59	104,4	108,69	106,44	**1,73**
YDR034W	YDR034W	YDR034W identified by SAGE expression analysis	514,24	600,11	556,9	929,75	1000,42	963,46	**1,73**
GPH1	YPR160W	Glycogen phosphorylase	65,66	83,56	74,41	117,81	137,6	127,68	**1,72**
YBL048W	YBL048W	YBL048W hypothetical protein	228,44	306,24	267,1	368,94	547,25	458,24	**1,72**
YKL071W	YKL071W	YKL071W weak similarity to A.parasiticus nor-1 protein	296,81	169,24	232,93	503,5	293,38	398,4	**1,71**
FUN34	YNR002C	Putative transmembrane protein	104,14	118,15	111,04	189,69	190,45	190,11	**1,71**
AMS1	YGL156W	Vacuolar alpha mannosidase	98,95	114,7	106,93	169,98	194,86	182,42	**1,71**
YMR196W	YMR196W	YMR196W hypothetical protein	76,06	133,12	104,57	130,8	223,09	176,91	**1,69**
RPP1B	YDL130W	Ribosomal protein P1B (L44') (YP1beta) (Ax)	280,58	380,39	329,8	502,77	610,39	556,68	**1,69**
RNY1	YPL123C	Ribonuclease from the T2 family of ribonucleases	51,02	60,03	55,49	80,17	104,73	92,46	**1,67**
UBP11	YKR098C	Ubiquitin-specific protease	52,95	48,32	50,51	102,8	65,38	84,02	**1,66**
NMR047C	NMR047C	Non-annotated SAGE orf Found reverse in NC_001145 between 623382 and 623516 with 100% identity.	172,38	165,53	169,26	279,33	282,99	280,93	**1,66**
YDL110C	YDL110C	YDL110C hypothetical protein	82,75	123,5	102,96	139,65	202,58	170,96	**1,66**
YMR181C	YMR181C	YMR181C similarity to YPL229w	339,17	299,27	319,48	495,11	558,76	526,82	**1,65**
YPL014W	YPL014W	YPL014W hypothetical protein	245,91	213,59	230,61	387,14	376,74	381,16	**1,65**
MOH1	YBL049W	YBL049W hypothetical protein	174,18	204,47	189,11	309,49	314,78	312,49	**1,65**
YLR149C	YLR149C	YLR149C hypothetical protein	57,05	115,46	86,24	120,77	161,36	141,02	**1,64**
YFLWDELTA	YFLWDELTA	YFLWDELTA5 Ty1 LTR	34,01	23,93	28,95	52,69	42,23	47,46	**1,64**
gGR07	gGR07	Saccharomyces cerevisiae chromosome VII, complete chromosome sequence. Found forward in NC_001139 between 547463 and 548462 with 100% identity.	160,07	249,96	204,92	343,8	323,49	333,4	**1,63**
YJL017W	YJL017W	YJL017W hypothetical protein	141,54	149,1	145,17	216,04	253,66	234,63	**1,62**
SHC1	YER096W	Sporulation-specific homolog of csd4	29,28	31,93	30,66	49,69	49,31	49,54	**1,62**
RAD54	YGL163C	DNA-dependent ATPase	70,64	67,61	69,39	114,45	110,67	112,34	**1,62**
NGR122W	NGR122W	Non-annotated SAGE orf Found forward in NC_001139 between 1012179 and 1012379 with 100% identity.	341,35	304	322,69	517,98	528,01	522,56	**1,62**
YMR090W	YMR090W	YMR090W strong similarity to B. subtilis conserved hypothetical protein yhfK	251	306,02	278,66	365,1	532,65	448,58	**1,61**
PEP12	YOR036W	Integral membrane protein\; c-terminal TMD\; located in endosome	60,96	69,64	65,04	96,03	113,01	104,55	**1,61**

DCS2	YOR173W	YOR173W strong similarity to YLR270w	31,41	69,27	50,5	55,31	107,45	81,41	**1,61**
YOR338W	YOR338W	YOR338W similarity to YAL034c	112,69	137,01	124,65	193,03	208,46	200,72	**1,61**
YPRWDELTA	YPRWDELTA	YPRWDELTA17 Ty1 LTR	165,98	91,76	128,84	209,21	204,29	206,9	**1,61**
OSW2	YLR054C	YLR054C hypothetical protein	82,17	93,12	87,79	131,91	149,19	140,57	**1,6**
YBR085C	YBR085C	YBR085C hypothetical protein	271,85	252,68	263,26	395,66	446,74	420,74	**1,6**
PHM8	YER037W	YER037W strong similarity to hypothetical protein YGL224c	103,32	163,29	133,13	185,47	240,92	213,15	**1,6**
TOS5	YKR011C	YKR011C hypothetical protein	118,54	163,3	140,87	218,15	230,3	224,13	**1,59**
AAD16	YFL057C	YFL057C strong similarity to aryl-alcohol dehydrogenases	988,48	1002,09	993,63	1764,47	1393,58	1579,2	**1,59**
YRO2	YBR054W	Homolog to HSP30 heat shock protein YRO1 (S. cerevisiae) 7	313,99	551,54	433,65	526,45	840,32	683,04	**1,58**
CMK2	YOL016C	Calmodulin-dependent protein kinase	270,13	299,74	284,47	473,08	417,68	445,44	**1,57**
INH1	YDL181W	ATPase inhibitor	193,2	229,76	211,31	255,06	406,83	330,88	**1,57**
YFR017C	YFR017C	YFR017C hypothetical protein	79,17	114,92	97,01	115,27	189,89	152,57	**1,57**
GTT1	YIR038C	Glutathione transferase	773,04	845,14	811,03	1249,15	1297,8	1273	**1,57**
NCA3	YJL116C	Involved in regulating expression of F0F1 ATPase subunits	995,07	1417,72	1206,26	1620	2137,85	1878,84	**1,56**
PHR1	YOR386W	Photolyase	22,32	36,18	29,12	41,01	50,22	45,56	**1,56**
PRX1	YBL064C	Similar to thiol-specific antioxidant enzymes such as rehydrin\/peroxiredoxin	171,99	274,82	223,3	284,89	410,84	347,75	**1,56**
GPD1	YDL022W	Glycerol-3-phosphate dehydrogenase	782,25	1145,29	963,3	1366,85	1643,44	1504,15	**1,56**
NTH1	YDR001C	Neutral trehalase	156,52	180,09	168,41	242,15	284,06	263,3	**1,56**
PNC1	YGL037C	Pyrazinamidase and nicotinamidase	535,05	959,55	747,64	895,74	1434,92	1165,55	**1,56**
SOL4	YGR248W	Similar to SOL3	36,92	49,86	43,72	50,31	86,63	68,41	**1,56**
YCR007C	YCR007C	YCR007C strong similarity to subtelomeric encoded proteins	101,04	101,64	101,33	141,85	173,59	157,82	**1,56**
GTT2	YLL060C	Glutathione transferase	632,99	845,7	738,83	1009,5	1275,14	1141,8	**1,55**
MSC1	YML128C	YML128C C-terminal part starting with aa 262 cause growth inhibition when overexpressed	106,4	134,13	120,32	142,77	230,38	186,49	**1,55**
GSP2	YOR185C	GTP binding protein, almost identical to Gsp1p	466,95	634,59	550,37	854,53	856,72	855,69	**1,55**
GUP2	YPL189W	Putative active glycerol transporter	29,86	31,55	30,56	51,49	43,4	47,28	**1,55**
NDL041C	NDL041C	Non-annotated SAGE orf Found reverse in NC_001136 between 34050 and 34184 with 100% identity.	109,07	114,44	111,49	189,01	157,2	173,22	**1,55**
HSP12	YFL014W	12 kDa heat shock protein	217,95	289,95	254,13	336,82	452,65	394,62	**1,55**

gGR07	gGR07	*Saccharomyces cerevisiae* chromosome VII, complete chromosome sequence. Found forward in NC_001139 between 548463 and 549462 with 100% identity.	170,88	245,49	208,26	333,87	310,88	322,58	**1,55**
TPO2	YGR138C	YGR138C similarity to multidrug resistance proteins	166,99	144,41	157,18	222,53	261,44	241,92	**1,54**
YLR297W	YLR297W	YLR297W weak similarity to Vibrio vulnificus VvpC protein	553,18	587,34	568,9	860,35	881,14	871,17	**1,53**
YOR385W	YOR385W	YOR385W strong similarity to hypothetical protein YMR316w	375,24	433,07	404,87	596,76	639,69	618,71	**1,53**
GPG1	YGL121C	YGL121C hypothetical protein	268,62	446,49	357,51	425,37	671,25	548,35	**1,53**
PET10	YKR046C	YKR046C hypothetical protein	863,55	1150,74	1006,15	1340,47	1713,63	1527,11	**1,52**
GON2	YLL033W	YLL033W hypothetical protein	66,7	53,39	60,32	99,1	84,28	91,69	**1,52**
BOP2	YLR267W	Bypass of PAM1	167,81	243,63	205,67	264,34	358,96	311,64	**1,52**
NNL034W	NNL034W	Non-annotated SAGE orf Found forward in NC_001146 between 452136 and 452276 with 100% identity.	31,98	41,34	36,56	50,28	60,88	55,71	**1,52**
YDL085C	YDL085C	YDL085C identified by SAGE	481,11	482,5	481,54	735,04	725,37	730,5	**1,52**
XBP1	YIL101C	Transcriptional repressor	77	104,32	90,81	134,42	142,68	138,43	**1,52**
YMR107W	YMR107W	YMR107W hypothetical protein	33,07	39,96	36,56	48,98	61,35	55,05	**1,51**
YBLCSIGMA1	YBLCSIGMA1	YBLCSIGMA1 Ty3 LTR	68,36	44,31	56,17	83,26	87,13	84,91	**1,51**
YLR327C	YLR327C	YLR327C strong similarity to Stf2p	151,7	250,95	201,28	229,64	375,61	302,62	**1,5**
UGA2	YBR006W	Succinate semialdehyde dehydrogenase	133,7	148,36	141,38	236,21	188,44	212,36	**1,5**
gNL04	gNL04	*Saccharomyces cerevisiae* chromosome XIV, complete chromosome sequence. Found forward in NC_001146 between 164130 and 165129 with 100% identity.	47,52	58,8	52,9	42,74	27,5	35,17	**-1,5**
YJL217W	YJL217W	YJL217W hypothetical protein	555,99	700,71	628,46	370,82	459,63	415,26	**-1,51**
PIR1	YKL164C	Protein containing tandem internal repeats	1935,63	2221,12	2076,99	1339,87	1412,16	1379,32	**-1,51**
TT(UGU)P	TT(UGU)P	TT(UGU)P tRNA-Thr	39,94	44,49	41,52	29,02	26,1	27,5	**-1,51**
RDN37-1	RDN37-1	RDN37-1 35S ribosomal RNA	313,34	182,22	247,86	248,32	78,83	163,55	**-1,52**
YPL158C	YPL158C	YPL158C weak similarity to human nucleolin	397,9	461,67	429,61	260,28	290,22	275,04	**-1,56**
PCL9	YDL179W	Cyclin	177,36	208,62	192,74	95,28	135,31	115,37	**-1,67**
HO	YDL227C	Homothallic switching endonuclease	166,65	264,67	215,74	126,06	131,88	128,15	**-1,68**
AMN1	YBR158W	Chromosome STability	1794,53	1868,29	1831,94	1063,85	1092,53	1079,39	**-1,7**

gAR03	gAR03	*Saccharomyces cerevisiae* chromosome I, complete chromosome sequence. Found forward in NC_001133 between 164789 and 165788 with 100% identity.	23,07	26,03	24,42	14,55	13,89	14,35	**-1,7**
gAR04	gAR04	*Saccharomyces cerevisiae* chromosome I, complete chromosome sequence. Found forward in NC_001133 between 210649 and 211648 with 100% identity.	29,75	19,08	24,27	17,24	11,4	14,22	**-1,71**
PRY3	YJL078C	Similar to plant PR-1 class of pathogen related proteins	2248,28	2408,09	2332,99	1337,91	1349,53	1344,61	**-1,74**
DSE1	YER124C	YER124C weak similarity to Dictyostelium WD40 repeat protein 2	187,53	151,42	169,54	92,61	102,39	97,45	**-1,74**
YGL028C	YGL028C	Soluble cell wall protein	803,7	790,73	797,24	415,71	469,19	442,02	**-1,8**
DSE3	YOR264W	YOR264W hypothetical protein	147,23	173,41	160,17	79,62	94,27	87,07	**-1,84**
NER034C	NER034C	Non-annotated SAGE orf Found reverse in NC_001137 between 407032 and 407214 with 100% identity.	1316,27	1055,99	1188,1	670,25	519,09	593,91	**-2**
